# The Role of *TCOF1* Gene in Health and Disease: Beyond Treacher Collins Syndrome

**DOI:** 10.3390/ijms22052482

**Published:** 2021-03-01

**Authors:** Małgorzata Grzanka, Agnieszka Piekiełko-Witkowska

**Affiliations:** Department of Biochemistry and Molecular Biology, Centre of Postgraduate Medical Education, 01-813 Warsaw, Poland

**Keywords:** *TCOF1*, treacle, nucleolus, Treacher Collins syndrome, ribosome biogenesis, DNA damage response, DDR, cancer

## Abstract

The nucleoli are membrane-less nuclear substructures that govern ribosome biogenesis and participate in multiple other cellular processes such as cell cycle progression, stress sensing, and DNA damage response. The proper functioning of these organelles is ensured by specific proteins that maintain nucleolar structure and mediate key nucleolar activities. Among all nucleolar proteins, treacle encoded by *TCOF1* gene emerges as one of the most crucial regulators of cellular processes. *TCOF1* was initially discovered as a gene involved in the Treacher Collins syndrome, a rare genetic disorder characterized by severe craniofacial deformations. Later studies revealed that treacle regulates ribosome biogenesis, mitosis, proliferation, DNA damage response, and apoptosis. Importantly, several reports indicate that treacle is also involved in cancer development, progression, and response to therapies, and may contribute to other pathologies such as Hirschsprung disease. In this manuscript, we comprehensively review the structure, function, and the regulation of *TCOF1*/treacle in physiological and pathological processes.

## 1. Introduction

Many of the crucial cellular processes that play an important role in proper functioning of the body occur in the nucleoli—small nuclear substructures devoid of a surrounding membrane [1,2,3]. Nucleoli are formed around short arms of five acrocentric chromosomes (13, 14, 15, 21, and 22), encoding 200–400 copies of actively transcribed rRNA genes, creating the nucleolus organizer regions (NORs) [1,2,4]. The formation of the nucleoli starts at the end of mitosis (telophase) when transcription of rDNA is initiated in NORs. The transcription and processing of rRNA is continued throughout early G1 phase, resulting in the formation of dense fibrillary component (DFC), which corresponds to the transcription of 47S rRNA. The following recruitment of late processing complexes around DFC leads to the formation of granular component (GC), which corresponds to the late steps of rRNA processing and results in the formation of large ribosome subunits. The mature nucleolus is composed of three substructures: the fibrillar center (FC), which confines the rDNA genes; DFC; and GC. At the beginning of prophase, rDNA transcription is continued; however, the processing proteins initially localized in DFC and GC leave the nucleolus, leading to the disruption of rRNA processing. At the end of prophase, transcription of rDNA shuts down and the nucleolus is disassembled [5].

The nucleolus is often referred to as a “plurifunctional” organelle [6]. Apart from its primary function, the biogenesis of ribosomes, the nucleolus is also involved in cell cycle progression, stress sensing, and DNA damage response. All these nucleolar activities are mediated by multiple proteins that ensure proper nucleolar structure and function [1,7,8,9,10]. One of the crucial proteins involved in most of the key nucleolar functions is treacle phosphoprotein encoded by the *TCOF1* gene.

*TCOF1* was initially discovered as a gene involved in Treacher Collins syndrome, a rare genetic disorder characterized by severe craniofacial deformations [11,12]. To date, the molecular basis of Treacher Collins syndrome, including the specific role of *TCOF1* mutations, have been extensively studied [13,14,15]. However, recent studies provide evidence that treacle regulates key cellular processes affecting not only development of facial skeleton, but also contributing to other pathological processes including cancer. In this manuscript, we comprehensively review the structure, function, and regulation of *TCOF1*/treacle both in physiological and pathological processes.

## 2. *TCOF1* Gene and Transcripts

The *TCOF1* gene (treacle ribosome biogenesis factor 1) was mapped in 1996 [11], and a year later the full sequence of its coding regions was published by Wise et al. [12]. *TCOF1* has been localized to the long arm of chromosome 5, at the 5q32-33.3 locus between the *CSF1R* and *SPARC* genes [11,16,17]. The human and mouse promoters of *TCOF1* gene include several predicted binding sites of transcription factors (e.g., c-myb, CCAAT, Zfp161, Sp1/Sp3, and AP2α), however, their functional significance is disputable [18]. 

Initially, it was assumed that the *TCOF1* gene is composed of 26 exons [12,19], but studies published in 2004 [20] indicated the presence of two additional exons: the 231 bp exon 6A (located between exons 6 and 7), and the 108 bp exon 16A (located between exons 16 and 17). The primary transcript of *TCOF1* is alternatively spliced into multiple mRNA isoforms (Figure S1), of which only a few contain exon 16A, while the vast majority includes exon 6A [20]. Importantly, exon 6A is preceded by pyrimidine-rich sequences, which are typical splicing acceptors. The full-length treacle protein is a product of translation of mRNA isoform containing exon 6A [20]. 

## 3. Structure and Localization of the Treacle Protein

**Figure 1:**
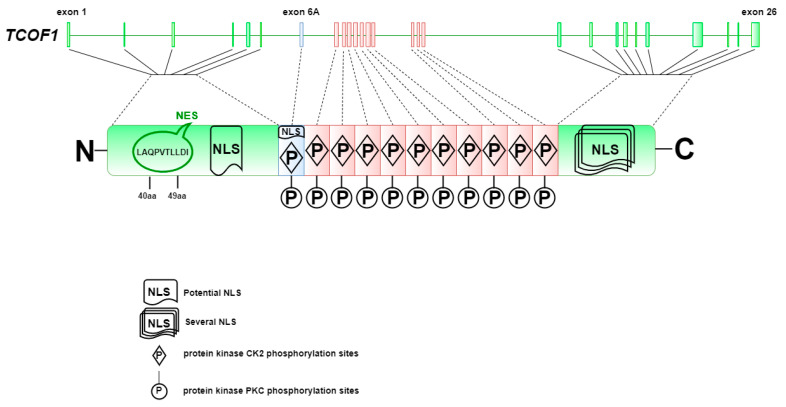
The structure of the major treacle isoform including amino acids encoded by exon 6A. The exon location was retrieved from Ensembl (https://www.ensembl.org), transcript ID: ENST00000377797.7, accessed on 11 May 2020. The N-terminus is encoded by six exons and contains nuclear export signal (NES) and potential nuclear localization signal (NLS) regions. The central domain encoded by exons 6A-16 consist of 11 repetitive motifs that are phosphorylated by CK2 and protein kinase C (PKC). The motif encoded by exon 6A has an additional NLS region. The *C*-terminus is encoded by exons 17–26 and contains several NLS regions in exons 23, 24 and 25 [12].

Treacle is a 1488-amino acid (aa), highly phosphorylated nucleolar protein with the predicted molecular weight of 152 kDa and a three-domain structure with unique *N*- and *C*-termini [20,21] (Figure 1). In SDS-PAGE, treacle migrates as a 220 kDa band [22]. The prediction of the originally cloned treacle cDNA sequence suggested a smaller protein composed of 1411 amino acids and molecular weight of 144 kDa [19,23]. Similar to other nucleolar phosphoproteins, treacle is a low-complexity protein [12,19]. Its structure, in particular the 10 repeat units of serine clusters, which are separated by alanine, lysine, proline, and glutamic acid [12,19,22], resembles the Nopp140 protein [12]. The key similarities between the two proteins include their low complexity in terms of amino acid composition and the repetitive basic and acidic regions, with the latter including multiple CK2 phosphorylation sites. Both proteins share several nuclear localization signals as well as two more similar regions, revealing 21% identity and 35% similarity [12]. Despite the homologous structures, treacle protein (in contrast to Nopp140) does not localize to Cajal bodies, the place where snRNAs, telomerase RNA, or histone RNA are processed [22,24].

The *N*-terminal treacle region contains the LAQPVTLLDI sequence motif, possibly acting as a nuclear export signal (NES) [20], located between 40 and 49 aa, as well as the nuclear localization signal (NLS) located in a region of 74–77 aa [12]. The *N*-terminus also contains several serine and threonine residues, which are phosphorylated by protein kinase CK2 and polo-like kinase 1 (PLK1), and mediate interactions with treacle-binding partners PLK1 and NBS1 [21,25]. It was also found that phosphorylation by Cdk1/Cyclin B1 facilitates treacle phosphorylation by PLK1 [25]. The central treacle domain contains numerous CK2 and PKC (protein kinase C) phosphorylation motifs, while the *C*-terminal region is equipped with several potential NLSs located between 1362 and 1482 amino acids (1285–1405 aa in the shorter predicted protein isoform) (Figure 1) [26]. A similar NLS signal is encoded by exon 6A, however, due to the lack of this exon in some transcript isoforms, this NLS does not seem to be necessary for the correct treacle localization, but it may be important for its interaction with other nucleolus components [20]. Treacle is also phosphorylated by ATM kinase, possibly at the 17 SQ/TQ motifs dispersed in the central and *C*-terminal protein regions [27].

During interphase, treacle localizes to the nucleolus, while during mitosis, it binds the centrosomes and kinetochores, which reflects its role as a mitotic regulator [13], further discussed below. In the nucleolus, treacle localizes to the dense fibrillary components in which rDNA transcription occurs [22], interacting with the key elements of rDNA transcription complex, including UBF (upstream binding factor) and RNA pol I (RNA polymerase I) [28].

## 4. The Role of *TCOF1*/Treacle in Physiological Processes

Treacle is widely distributed throughout most human tissues and organs. Its expression occurs both in adult and embryonic tissues [11]. During mouse development, the expression of *TCOF1* peaks in E8.5-9.5 embryos and is particularly pronounced in the first pharyngeal arch (which develops into the mandible and maxilla), as well as in the neuroepithelium and developing brain [29,30]. Consistently with its expression patterns, treacle contributes to the development of the facial skeleton [29,30] as well as the central and enteric nervous systems [25,31]. From the molecular perspective, treacle is necessary for proper rDNA transcription [28], as well as biogenesis and modifications of ribosomes, contributing to the translation patterns in the cell [32]. At the cellular level, treacle contributes to the regulation of mitosis and proliferation [13,25,30], and protects against apoptosis induced by oxidative stress [13]. The mechanistic details of cellular processes regulated by treacle are discussed below.

### 4.1. The Role of TCOF1 in Ribosome Biogenesis and Function

**Figure 2:**
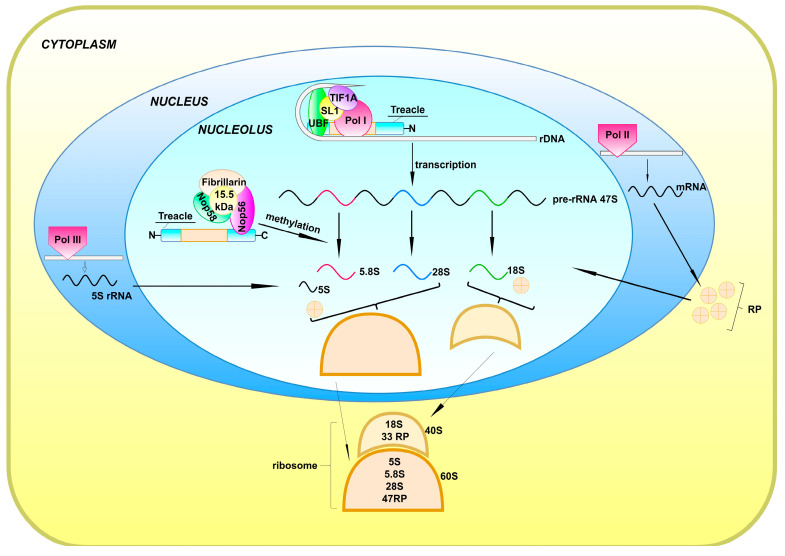
Ribosome biogenesis begins in the nucleolus and is continued during the transport via the nucleoplasm and the final maturation steps that occur in the cytoplasm. The treacle protein recruits the pre-initiation complex (UBF, SL-1, Pol I with associated TIF1A) to the rDNA promoter region [40]. The produced 47S pre-rRNA undergoes post-transcriptional processing (including methylation and possibly pseudouridylation), involving treacle, which binds ribonucleoprotein methylation complex composed of Nop56, fibrillarin, Nop58, and 15.5 kDa protein [32,41]. As a result of the post-transcriptional processing, 5.8S, 18S, and 28S RNAs are generated. The residual ribosome components are synthesized in the nucleoplasm and cytoplasm—Pol III transcribes a gene encoding 5S RNA, which is then transported to the nucleolus; Pol II transcribes genes encoding ribosomal proteins, resulting in mRNAs that are transported to the cytoplasm where translation of ribosomal proteins (RPs) takes place. RPs are then translocated to the nucleolus, where ribosome subunits are formed—the large one (60S), composed of 5S, 5.8S, 28S and 47RP, and the small one (40S), composed of 18S and 33RP. The ribosome subunits migrate to the cytoplasm where the mature ribosome is assembled [35].

The biogenesis of ribosomes is one of the most important processes in the cell. It is responsible for as much as 95% of total transcription and consumes more than 60% of cellular energy [33,34]. The ribonucleic components of ribosomes consist of 18S rRNA (which is a backbone of small ribosome subunit, 40S), as well as 5S, 5.8S, and 28S rRNAs (which form the large ribosome subunit, 60S). Most of these transcripts are derived from posttranscriptionally processed large 47S pre-rRNA, resulting in 5.8S, 18S, and 28S rRNAs. The gene encoding 5S rRNA is independently transcribed by polymerase III [35]. The biogenesis of ribosomes occurs in the nucleoli [1,2] and consists of three stages: (1) transcription of rDNA into pre-rRNA, (2) post-transcriptional pre-rRNA processing, and (3) ribosome assembly [4,36,37,38]. The first stage begins in the nucleolus with the formation of the pre-initiation complex (PIC) around the rDNA promoter region [2]. It consists of UBF, SL1, and RNA Pol I, which associates with TIF1A (transcription initiation factor 1A) [2,39] (Figure 2).

Treacle plays a crucial role in ribosome biogenesis by binding and recruiting Pol I, UBF, and Nopp140 to the rDNA promoter [40]. These interactions are mediated by different treacle domains, with the central repeated domain (amino acids 526–961) binding to Pol I, and the *C*-terminal domain interacting with UBF (treacle amino acids 1294–1488), Nopp140 (amino acids 1294–1488), and rDNA (amino acids 1294–1488) [40]. The interaction of treacle with rDNA promoter was confirmed by chromatin immunoprecipitation [40,41]. Silencing of treacle leads to dispersion of Pol I and UBF from the nucleolus [40], and results in inhibition of rDNA transcription [21,28].

Treacle acts as an activator of UBF, an important regulator of rDNA transcription [28]. UBF actions are bimodal—on one hand, it mediates the assembly of the PIC at the promoter regions by binding to SL1, while on the other hand, it induces changes in chromatin condensation by replacing histone complexes throughout the rDNA-transcribed regions and enabling recruitment of other transcription factors [42]. Treacle directly interacts with UBF, enabling its function in rDNA transcription [21,28].

During the second stage of ribosome biogenesis, treacle regulates post-transcriptional pre-rRNA modifications, including methylation [41] and, possibly, pseudouridylation [32]. 2′-*O*-Methylation of pre-rRNA is a process that protects RNA against hydrolysis, affects flexibility of ribonucleic acid strand, and regulates translational programs in the cell [43]. Treacle regulates this pre-rRNA modification by interacting with Nop56, a key component of the ribonucleoprotein methylation complex, and probably acting as a scaffold for other protein components. Nucleotide modifications of rRNA are guided by C/D and H/ACA snoRNAs. The nucleolar localization and proper functioning of snoRNAs is ensured by their complexing proteins. Specifically, C/D snoRNA associates with SNU13, NOP56/NOP58, and fibrillarin, while H/ACA snoRNA forms a complex with NHP2, NOP10, GAR1, and pseudouridine synthase DKC1 [44]. Insufficient treacle expression (induced by siRNA silencing or due to *TCOF1* haploinsufficiency in mice) attenuates pre-rRNA methylation [41] (Figure 2).

#### 4.1.1. The Role of Treacle Ubiquitilation

Treacle interacts with KBTBD8 [32], which is an adaptor for CUL3, a scaffold protein for an E3 ubiquitin ligase complex [45]. Binding of KBTBD8 to treacle and NOLC1, another nucleolar protein, induces their monoubiquitylation, with β-arrestin acting as a cofactor, leading to their stabilization. The resulting treacle/NOLC1 complex serves as a platform recruiting Pol I, as well as the enzymes involved in rRNA pseudouridylation (the H/ACA complex), and controlling maturation and modification of the small ribosomal subunit (the SSU processome) [32]. During neural crest development, this CUL3^KBTBD8^-treacle-induced mechanism leads to the global reprogramming of translation, delaying accumulation of specific CNS (central nervous system)-precursor proteins until pluripotent stem cells reach proper maturation state [32]. The CUL3^KBTBD8^-mediated stabilization of treacle is regulated by phosphorylation of the latter. Specifically, phosphorylation of treacle by protein kinase CK2 promotes its interaction with CUL3^KBTBD8^, while its dephosphorylation by PP1 and PP2A phosphatases prevents binding and monoubiquitilation by CUL3^KBTBD8^. Neural crest specification and CUL3^KBTBD8^-dependent monoubiquitilation require CK2 multi-site substrate phosphorylation [46]. Mechanistically, treacle contains 10 motifs, which can be phosphorylated by CK2 and then independently bound by KBTBD8. This in turn enables treacle monoubiquitilation at sites that are located in the proximity to CK2 motifs. This regulation plays an important role during neural crest maturation as expression of CK2 slowly increases, while expression of PP1A gradually decreases during neural crest development. The multisite interaction between phosphorylated treacle and KBTBD8 ensures optimal substrate recognition by CUL3^KBTBD8^ and allows cells to react to small changes in the activity of CK2. Another essential substrate of CUL3^KBTBD8^ is NOLC1, which similarly to treacle is phosphorylated by CK2 at multiple sites [46]. Since NOLC1 and treacle act in a common pathway, depletion of any of them results in misspecification of the neural crest and increased abundance of CNS precursors [32].

Remarkably, CK2-mediated phosphorylation is also required for treacle interaction with NBS1 (Nijmegen breakage syndrome protein 1) in a mechanism regulating rRNA transcription in response to DNA damage [21], which is further discussed below.

### 4.2. Treacle and the Response to DNA Damage

Every day, each cell of our body acquires up to 100,000 DNA changes, which arise spontaneously or appear as a result of the influence of environmental factors [47]. To achieve the genomic integrity, these damages must be repaired by the cell. Deficiencies in DNA damage-repairing systems contribute to serious disorders such as cancers or inflammatory and neurodegenerative diseases [48]. The genes encoding rRNA are particularly prone to damage, as rDNA is intrinsically unstable due to erroneous recombination occurring between rDNA sequences from different chromosomes in which rRNA genes are located [27]. Therefore, in response to DNA damage, cells create a signal transduction pathway called the DNA damage response (DDR) [47,48]. The DDR involves many repair mechanisms depending on the type of DNA damage [47]. Single-strand DNA lesions can be reconstructed by (i) base excision repair (BER), in which DNA glycosidase recognizes the damaged base, or (ii) nucleotide excision repair (NER), which recognizes nucleotide changes that cause helix deformation [47,48]. While the NER and BER are relatively easy to execute, due to the presence of the second, untouched strand that can serve as a “template” for the repair process [47], double-strand breaks (DSB) are particularly dangerous for the cell [48]. The DSB repair mechanisms involve two processes: NHEJ (non-homologous end joining) or HR (homologous recombination) [48,49]. NHEJ is usually inaccurate and error-prone due to change or loss of several nucleotides that can occur during the repair process, resulting in so-called “information scars” [47,50]. Despite this, the repair usually starts with NHEJ and only when the DNA damage turns out to be more complex does the cell then begin the HR repair pathway [51]. HR occurs not only during DNA repair, but also ensures proper segregation of homologous chromosomes during meiosis [49]. HR is a very precise DSB repair pathway; however, it is limited to the S and G2 phases of the cell cycle because it requires the presence of homologous sister chromatid [47]. The HR process starts with a deep resection of DNA ends, which involves the heterotrimeric MRN complex, consisting of MRE11, RAD50, and NBS1 proteins [48]. Following binding and stabilization of DSB ends, MRN recruits ATM and ATR kinases, which trigger DNA damage-signaling pathways and induce transcriptional silencing. Attenuation of transcription reduces the energy usage and prevents the collision between the complexes catalyzing transcription and repair [52]. Transcriptional silencing results in reorganization of the nucleoli due to migration of rDNA–protein complexes from nucleolar centers towards peripheries, where they form specific structures called the nucleolar caps. The accumulation of DSB rDNA in the nucleolar caps facilitates the access and functioning of protein complexes that mediate repair of damaged rDNA [53].

**Figure 3:**
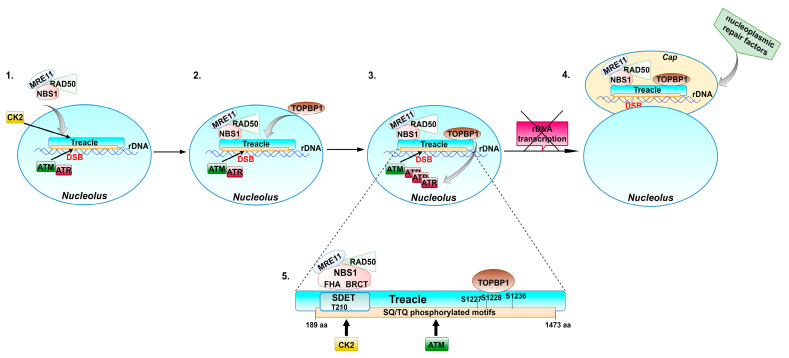
The proposed role of treacle in response to rDNA damage. Treacle facilitates DNA damage response (DDR) mechanisms involving ATM and ATR kinases by recruiting their key adaptor proteins, NBS1 and TOPBP1, respectively. (**1**) Following rDNA damage, ATM and ATR kinases are initially activated at the sites of rDNA breaks (DSB) independently of treacle [56]. ATM- and CK2-catalyzed phosphorylation enables treacle to recruit NBS1, which mediates ATM interactions with MRN complex [21,27,55]. (**2**) ATM-mediated phosphorylation of treacle enables recruitment of TOPBP1. The efficient TOPBP1 recruitment requires the presence of NBS1 [56]. (**3**) NBS1 and TOPBP1 localized at the sites of rDNA damage enable further recruitment and activation of ATR kinase, needed for repression of Pol I-catalyzed transcription [56]. (**4**) Inhibition of rDNA transcription enables formation of the nucleolar caps. The localization of damaged rDNA in nucleolar periphery facilitates recruitment of repair factors from the nucleoplasm [58]. (**5**) The scheme of treacle with depicted phosphorylation sites involved in interactions with NBS1 and TOPBP1. NBS1 interacts with treacle via FHA and BRCT domains [21,56].

Treacle plays a crucial role in the DDR mechanisms involving ATM and ATR kinases by recruiting their key adaptor proteins, NBS1 and TOPBP1, respectively (Figure 3). Proper assembly and localization of MRN complex depends on NBS1, which acts as a chaperone, ensuring the appropriate conformation of the MRE11–RAD50 complex [54]. Treacle interacts with NBS1 using a SDET motif located in the *N*-terminal domain, with CK2-phosphorylated threonine 210 as the main binding site. This CK2-mediated phosphorylation is a prerequisite for treacle-mediated binding and nucleolar translocation of NBS1 [21,55]. Remarkably, the recruitment of NBS1 to rDNA is also dependent on the ATM-mediated phosphorylation of SQ/TQ motifs, dispersed between 189 and 1473 amino acids of treacle [27], with particular involvement of phosphoserine S1199 [55]. Phosphorylation also regulates the interaction of treacle with TOPBP1 [56], a crucial regulator of ATR kinase activity [57]. Specifically, binding of TOPBP1 is mediated by phosphoserines (S1227, S1228, and S1236) located in the *C*-terminal treacle region [56]. S1236 is one of the SQ motifs recognized by the ATM kinase [56]. According to the recently published models [53,56], treacle is involved in a positive feedback mechanism, which leads to nucleolar accumulation of ATR, required for transcriptional repression in response to rDNA damage. Mooser et al. [56] proposed that following rDNA damage, ATM and ATR kinases are initially activated at the sites of rDNA breaks independently of treacle. In response to rDNA damage, treacle binds and recruits NBS1 to the nucleolus in an ATM phosphorylation- and CK2 phosphorylation-dependent manner [21,27,55]. On the other hand, ATM-mediated phosphorylation of treacle enables recruitment of TOPBP1 [56]. The efficient TOPBP1 recruitment requires the presence of NBS1 [56]. The presence of NBS1 and TOPBP1 at the sites of rDNA damage enables further recruitment and activation of ATR kinase, needed for repression of Pol I transcription [56]. Inhibition of rDNA transcription leads to fusion of FC and DFC, which migrate towards nucleolar peripheries to form nucleolar caps in which the repair of rDNA breaks takes place. The localization of damaged rDNA in nucleolar periphery enables recruitment of repair factors from the nucleoplasm [58].

### 4.3. TCOF1 and the Mitotic Regulation

The functional links between treacle and mitosis are illustrated by its changes in cellular localization at different mitotic phases. During the interphase, treacle localizes to the nucleolus [28], which reflects its involvement in the regulation of rRNA transcription. At the end of the prophase, rRNA transcription is halted and nucleoli disassemble, leading to the release of the nucleolar proteins [5]. This enables binding of the released treacle to centrosomes and kinetochores during prophase, prometaphase, and metaphase, with the following localization to the midzone in anaphase and the midbody in telophase [25]. Treacle directly regulates the formation of the mitotic spindle by acting as a scaffold for Polo-like kinase 1 (Plk1), a crucial regulator of mitosis [25]. Plk1 phosphorylates and regulates proteins involved in all key steps of mitosis, including those regulating the breakdown of the nuclear envelope, segregation of sister chromatids, formation of kinetochores, as well as proteins involved in metaphase-to-anaphase transition, and cytokinesis [59]. Treacle enables these Plk1 functions by providing a scaffold for its proper localization at the prophase and telophase. Interestingly, the mutual interaction of the two proteins results in sequential treacle phosphorylation, firstly by Cdk1/Cyclin B1, with the following additional phosphorylation by Plk1. Loss of treacle or inhibition of Plk-1 results in disorganization of the mitotic spindle and improper alignment or incomplete assemble of chromosomes at the metaphase plate. In consequence, treacle loss results in mitotic arrest and cell cycle progression delay [25]. Using mouse model, Sakai et al. demonstrated that both treacle and Plk1 are critical for proper neurogenesis of mammalian brain, providing mechanistic explanation for the neurodevelopmental disorders, including these associated with TCS such as microcephaly [25].

### 4.4. TCOF1 and Telomere Integrity

Treacle facilitates telomere replication by suppressing transcription of telomeres. Specifically, during the S phase, treacle leaves the nucleolus and is recruited to telomeres by interacting with TRF2, a sheltering protein. This in turn enables treacle-mediated suppression of Pol II elongation activity, resulting in attenuation of telomere transcription [60]. Eukaryotic telomeres are transcribed into long non-coding RNAs called TERRA, which can interfere with replication fork by forming R-loops, three-stranded hybrids composed of nascent RNA and DNA templates [61]. Treacle-mediated suppression of TERRA transcription prevents formation of the R-loops, their interference with replication fork, and fork stalling. In consequence, depletion of treacle in U2OS cells leads to an increased number of telomere-free ends and multiple telomere signals (MTS), synonymous with fragile telomere phenomena [60].

## 5. The Role of *TCOF1* in the Pathological Processes

**Figure 4:**
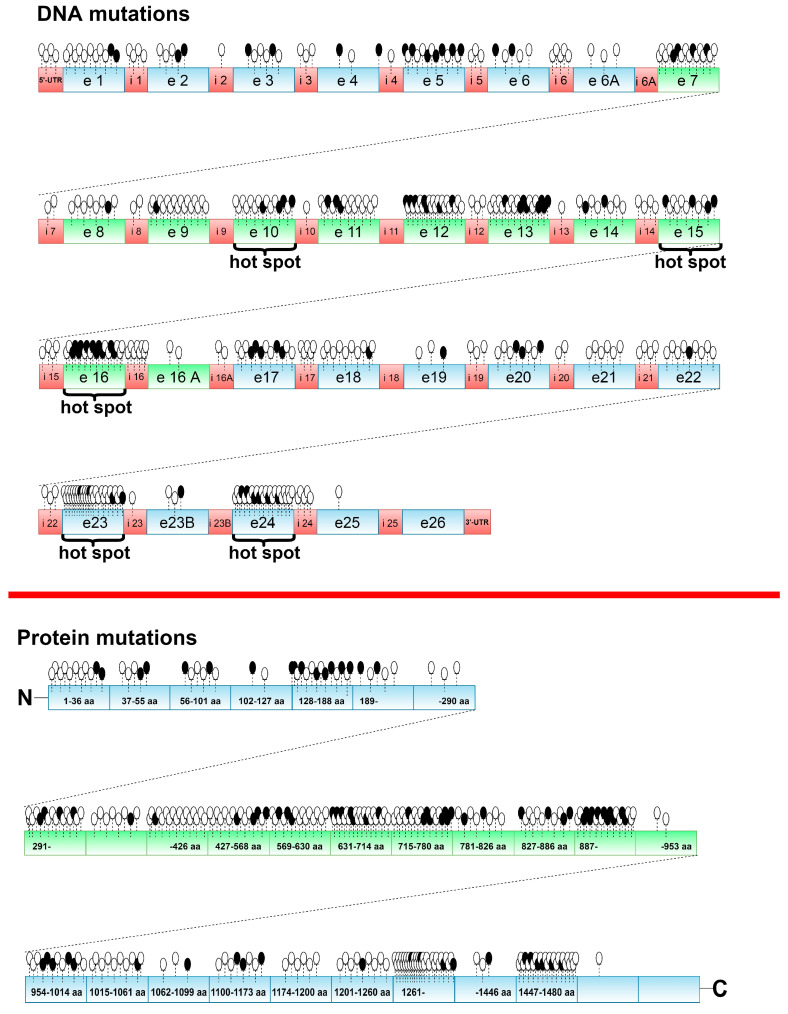
*TCOF1* polymorphisms and mutations detected in patients with Treacher Collins syndrome (TCS) and described thus far in the literature. The exonic/intronic localization of mutations (**upper panel**) refers to data provided in Table S1. The localization of the corresponding amino acids (**lower panel**) is based on NCBI Reference Sequence: NP_001128715.1. Each pin represents one genetic change. Black pins indicate changes that result in a truncated protein. Detailed characteristics of the mutations are provided in Table S1.

When we consider the crucial roles of treacle in biogenesis of ribosomes, DNA damage response, and mitotic regulation, it is not surprising that *TCOF1* dysfunction can lead to developmental disorders and can affect the functioning of mature cells. Strikingly, bi-allelic *TCOF1* inactivation has not been described thus far, suggesting possible embryonic lethality [28]. The ClinVar database (ncbi.nlm.nih.gov/clinvar; accessed on 2 February 2021) includes 253 sequence variants of the *TCOF1* gene, of which 81 have pathogenic clinical significance, 15 are described as likely pathogenic, 73 as likely benign, 62 as benign, 3 with conflicting interpretation, and 30 of uncertain significance. Regarding the resulting change in the gene product sequence, 44 variants cause sequence frameshift, 79 are classified as missense mutations, 20 as nonsense changes, 4 affect splice site, and 2 are located in the UTR. Variant types include 42 deletions, 29 duplications, 2 indels, 19 insertions, and 168 single-nucleotide polymorphisms (SNPs). The vast majority of *TCOF1* mutations are associated with Treacher Collins syndrome (TCS) (Table S1, Figure 4). The key pathologies linked with *TCOF1* dysfunction are discussed below.

### 5.1. Treacher Collins Syndrome

Treacher Collins syndrome (TCS) was described by Edward Treacher Collins in 1900, and further characterized by Franceschetti and Klein in 1949 [62,63]. It occurs in 1 of 50,000 live births and is most often inherited in an autosomal-dominant manner [13,15,64]. TCS is characterized by severe craniofacial defects, including facial bones hypoplasia, cleft palate, downwarding slant palpebral fissures, and external ear deformations, as well as hearing loss and microcephaly. Mental retardation is infrequent in TCS [13,15,63,65].

The mechanism underlying TCS has been well established on the basis of animal studies in mice and zebrafish models [14,30,63,66,67]. Although TCS can be also caused by *POLR1C* and *POLR1D* gene mutation, the vast majority (up to 93%) of TCS cases are associated with mutations of *TCOF1* [67,68,69,70]. The majority of these mutations are deletions, mostly causing premature termination codon, resulting in a truncated treacle protein or nonsense-mediated mRNA decay [12,71,72,73,74] (Table S1). A total of 17% of TCS cases are caused by 5bp deletions located in exon 24 [75]. Insertions [73] and duplications [71] also occur frequently, usually altering 1–41 bp in the coding regions of the gene [75]. The hotspot regions most commonly affected in TCS are *TCOF1* exons 10, 15, 16, 23, and 24, which account for more than a half of the pathogenic mutations [73]. In 60% of TCS cases, causative mutations are spontaneous and there seems to be no genotype–phenotype correlation [76].

**Figure 5:**
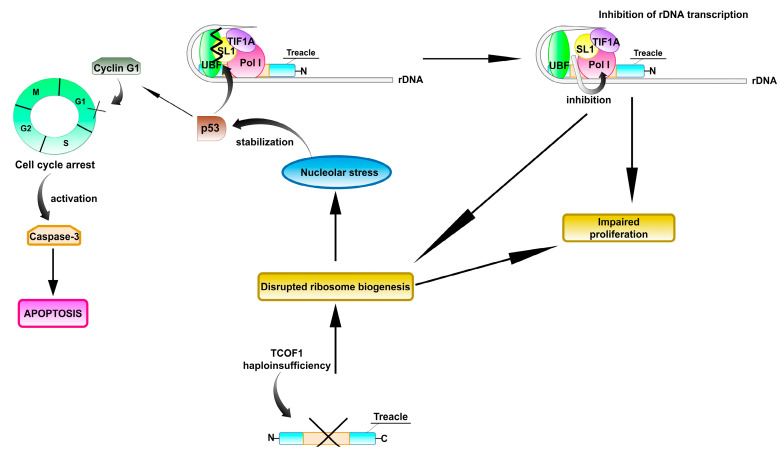
The model of *TCOF1* haploinsufficiency leading to TCS proposed by Jones et al. [82]. *TCOF1* haploinsufficiency leads to disruption of ribosome biogenesis, impaired proliferation, and apoptotic loss of neuroepithelial and neural crest cells. This mechanism is further boosted by p53 tumor suppressor, which is stabilized in response to the nucleolar stress. p53 prevents the interaction between SL1 (promoter selectivity factor 1) and UBF, leading to inhibition of RNA polymerase I activity and attenuation of rRNA transcription [83]. This in turn leads to the reduced ribosome biogenesis and attenuated proliferation. On the other hand, p53 activates the transcription of cyclin G1, which leads to cell-cycle arrest in G1 phase, with the following induction of apoptosis. The apoptotic loss of neural crest precursors leads to craniofacial abnormalities and TCS development. Importantly, inhibition of p53 function in mouse TCS embryos reduces the activation of cyclin G1 and consequently attenuates apoptosis, preventing TCS in *TCOF1*^+/−^ embryos. The above mechanism has been described by Jones et al. [82] as a mechanistic model describing the molecular background of TCS pathogenesis. Later studies revealed that treacle loss also results in dysfunction of Polo-like kinase 1 (Plk1), leading to mitotic arrest and cell cycle delay of developing brain neurons and contributing to the TCS neurodevelopmental disorders such as microcephaly [25]. Another postulated mechanism of *TCOF1* haploinsufficiency resulting in TCS could be lowered rRNA production and global reprogramming of cellular translation program [32] (see text for details).

During normal embryonic development, the expression of *TCOF1* is tightly regulated in a spatio and temporal manner, with pronounced expression in the cells of neural crest (NC), from which craniofacial skeleton is derived. One of the mechanisms by which *TCOF1* haploinsufficiency leads to TCS development is disruption of ribosome biogenesis leading to restriction of cell cycle, impaired proliferation, and apoptotic loss of cells of neuroepithelium and neural crest [30]. According to multiple studies, disruption of ribosome biogenesis is a strong inducer of p53 [77,78,79,80,81]. Jones et al. proposed that the nucleolar stress resulting from impaired ribosome biogenesis leads to stabilization of p53, which in turn activates the transcription of cyclin G1, leading to cell cycle arrest and induction of apoptosis [82]. Moreover, p53 activation can further boost the initial impairment of ribosome biogenesis triggered by *TCOF1* haploinsufficiency. Specifically, p53 can repress rDNA synthesis by binding to SL1 and blocking its interactions with UBF, thereby inhibiting Pol I transcription [82,83]. According to the model provided by Jones at al. [82], this contributes to the restriction of ribosome biogenesis and reduced proliferation of neuroepithelial and NC cells. The resulting deficiency of NC cells disables proper formation of facial bones, leading to the development of characteristic phenotypic features of TCS (Figure 5). Remarkably, Jones et al. demonstrated that chemical or genetic inhibition of p53 can prevent development of craniofacial abnormalities in a mouse TCS model [82].

The development of neuroepithelium in normal embryos is associated with highly oxidative state resulting from the intense cell proliferation and high metabolic activity [13,14]. The produced reactive oxygen species (ROS) are the major threat to DNA, capable of introducing breaks in DNA strands [47,48]. Due to its role in DNA damage response, treacle protects neuropithelial cells against ROS effects, while *TCOF1* loss in a mouse TSC model results in an increased DNA damage and caspase-3-induced apoptosis. Consistently with this observation, antioxidant prenatal treatment of *TCOF1-*haploinsufficient mouse embryos attenuates neuroepithelial apoptosis and ameliorates craniofacial abnormalities [14]. In a zebrafish model of TCS, treacle depletion is associated with the increased ROS level and induction of redox-responsive genes [84]. These effects of *TCOF1* haploinsufficiency were counteracted by Cnbp, a ROS-cytoprotective protein [84].

Another postulated mechanism of *TCOF1* haploinsufficiency resulting in TCS could be lowered rRNA production and global reprogramming of cellular translation program [32,41]. As mentioned above (Section 4.1.1) treacle interacts with NOLC1 to form a platform that couples RNA polymerase I with enzymes catalyzing rRNA modifications (methylation, pseudouridylation) and ribosome processing. Werner et al. proposed that the resulting pattern of rRNA and ribosome modifications affects interactions of ribosomes with the selected mRNAs, as well as proteins involved in the synthesis or degradation of specific mRNAs, hence influencing the final pool of proteins, which are translated. According to the proposed model, treacle prevents the accumulation of CNS proteins until neural crest cells reach the appropriate differentiation stage. Thus, loss of treacle may result in perturbation of this tightly coordinated sequence of the translation of specific groups of proteins, resulting in dysregulation of neural crest formation [32].

Despite the relatively well recognized role of treacle in the regulation of craniofacial development, the specific mechanisms, by which *TCOF1* mutations lead to TCS development are less well understood. Most *TCOF1* mutations associated with TCS lead to premature STOP codons, which should result in the production of a truncated protein and, consequently, the loss of its function. However, the truncated treacle protein was not detected in the fibroblasts and lymphoblasts of patients with TCS, while the level of full-length treacle in TCS patients’ cells does not differ from healthy controls [22]. In contrast, *TCOF1* transcript levels are significantly reduced in TCS patients compared with healthy controls [85]. It was proposed that the possible loss of treacle encoded by a mutated allele could be compensated by post-translational regulatory mechanism [85]. Similar discrepancies between the in vitro studies and ex vivo observations of TCS patients’ cells were found for mutations affecting the NLS sequences. Ectopic expression of treacle truncated at the *C*-terminus disables nucleolar targeting of the protein [40]. However, mislocalization of treacle and the other nucleolar proteins is not observed in fibroblasts of TCS patients [22]. It was suggested that the pathogenic effects of *TCOF1* haploinsufficiency could occur selectively during embryonic development, especially in the cells of 1 and 2 branchial arches, which require high and precisely regulated levels of treacle expression. The compensation mechanism could not be sufficient here, leading to the aberrances in neural crest maturation [22]. Remarkably, *TCOF1* mutations identified in TCS patients can affect amino acids involved in the interactions with Pol I, NOP56, UBF, Nopp144, and TOPBP1, or phosphorylation sites (Table S1), crucial for treacle functioning in ribosome biogenesis and DDR, suggesting another possible mechanism of treacle dysfunction in TCS.

### 5.2. The Role of TCOF1 in Cancer

The first suggestions on the possible role of *TCOF1* in tumorigenesis came from Jones et al. [82]. Their studies in murine TCS models revealed that *TCOF1* haploinsufficiency leads to the upregulation of p53 protein, thereby triggering apoptosis of neuroepithelial and neural crest cells [82]. The same study showed that treacle is required for cell cycle progression and its loss leads to the upregulation of cyclin G1, which arrests cells in G1 phase [82]. Jones et al. hypothesized that inhibition of treacle could rescue *TP53* haploinsufficiency, preventing tumor development. Although they did not verify this hypothesis experimentally, they noted that *TCOF1* deficiency could prevent or delay cancer development, consistently with the lack of data on cancer cases among TCS patients [82].

**Figure 6:**
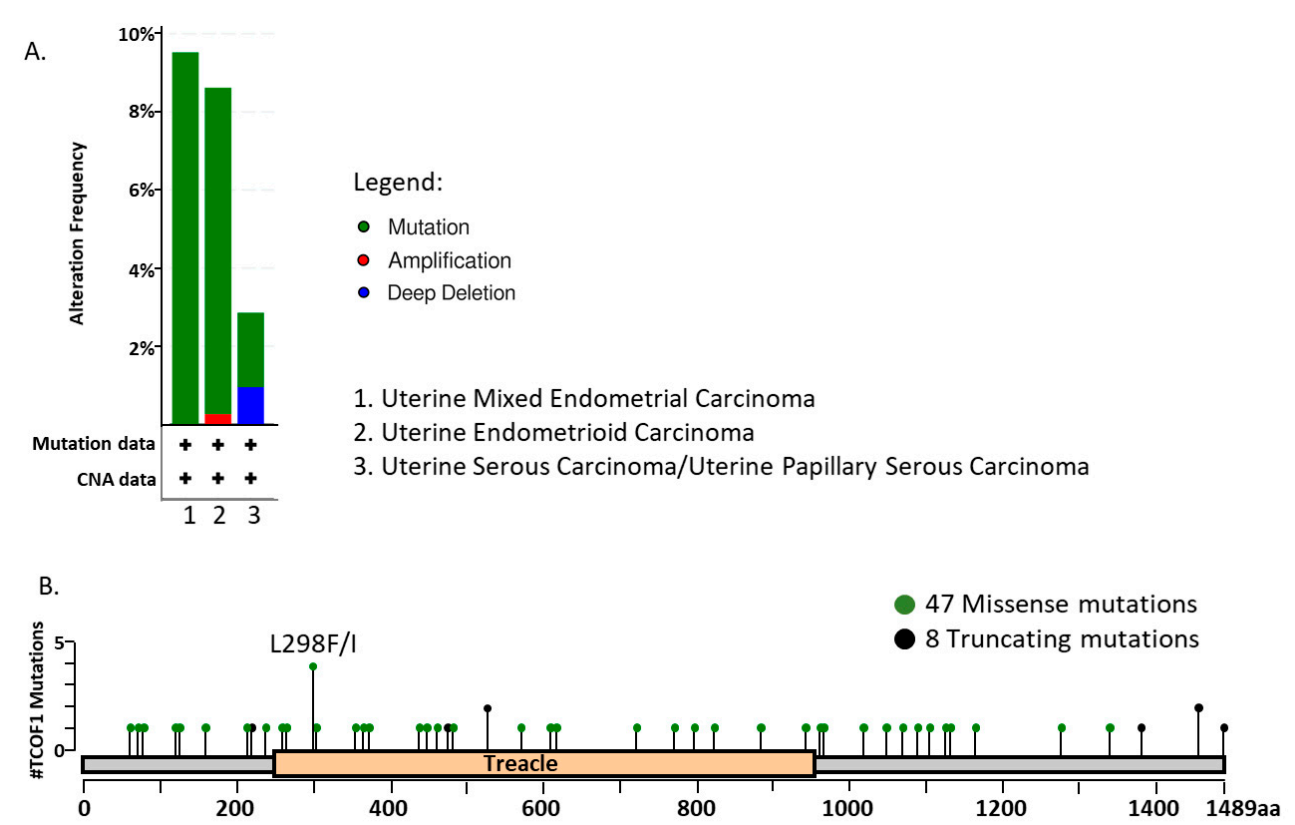
*TCOF1* mutations in uterine carcinoma. (**A**) The frequency of *TCOF1* alterations in 509 samples of uterine carcinomas, classified as uterine mixed endometrial carcinoma (*n* = 21), uterine endometrioid carcinoma (*n* = 383), and uterine serous carcinoma/uterine papillary serous carcinoma (*n* = 105). (**B**) Graphical representation of mutations identified in uterine carcinomas. In total, 55 *TCOF1* mutations were found in 509 samples of uterine carcinomas. The figure shows results of bioinformatic analysis of the publicly available transcriptomic data of The Cancer Genome Atlas, retrieved and analyzed using cBio platform (http://www.cbioportal.org/, accessed on 11 May 2020).

Surprisingly, the studies directly analyzing the role of *TCOF1* in cancer are limited. Analysis of transcriptomic data from >500 uterine carcinoma patients demonstrates that *TCOF1* alterations can be detected in nearly 10% of the analyzed cancer samples, with a clear hot spot mutation affecting codon 298 and the resulting substitution of leucine with isoleucine or phenylalanine (Figure 6). The significance of these mutations remains to be clarified; however, studies in cell lines derived from other tumor types suggest that *TCOF1* disruption can affect functioning of cancer cells. Large-scale transcriptomic analyses revealed that depletion of treacle in neuroblastoma cells affects the expression of genes involved in proliferation, apoptosis, cell cycle, differentiation, migration, and angiogenesis [86], suggestive of its global effect on cancer-related pathways. Furthermore, siRNA-mediated *TCOF1* silencing in cervical carcinoma HeLa cell line results in inhibition of rDNA transcription and attenuation of cell proliferation [28]. Interestingly, treacle regulates functioning of DDX21, an RNA helicase, which promotes gastric cancer proliferation and tumor growth [87]. Specifically, *TCOF1* silencing in HeLa cells leads to the nucleoplasmic relocation of DDX21, with concomitant loss of its binding to rDNA and Pol II target promoters [88]. Another procancerous feature of treacle is its ability to protect lung cancer cells against ROS [89], in accordance with the fact that cancer cells express proteins that protect them against oxidative stress [90].

As above mentioned, treacle is required for nuclear translocation and accumulation of NBS1, a crucial regulator of DNA damage responses [21,55]. Remarkably, NBS1 aberrances and the resulting inability of cells to respond to DNA damage contributes to chemoresistance and tumor development [91,92,93]. Treacle-mediated NBS1 accumulation in the nucleoli is crucial for silencing of rRNA transcription in response to DNA damage [21]. Since DDR dysfunction is closely associated with tumorigenesis [94] and chemoresistance [95], it may suggest that inappropriate treacle actions could disturb NBS1 functioning, thereby contributing to cancer development and progression as well as response to therapies. Furthermore, treacle also regulates sensitivity to radiotherapy. Enhanced *TCOF1* expression confers radioresistance of acinar progenitor cells of rat salivary glands [96]. Specifically, attenuation of *TCOF1* expression resulted in sensitization of progenitor cells to radiation [96]. Similarly, silencing of *TCOF1* in human osteosarcoma cells sensitized the cells to irradiation and cisplatin treatment [55]. This is in line with the fact that cells deficient in the ability to rapidly repair DNA are more prone to cisplatin-induced cell death [97]. These effects of *TCOF1* silencing may possibly be mediated by TOPBP1, since treacle regulates DNA damage response by recruiting TOPBP1 [56] while TOPBP1 expression confers radioresistance of osteosarcoma cells [56]. Interestingly, cisplatin cytotoxic effects are mediated by UBF, a binding partner of treacle. Specifically, cisplatin–DNA adducts act as a decoy for UBF, which induces its rapid displacement from rDNA sequences, leading to the inhibition of rRNA synthesis [98]. Since UBF is normally tightly bound by treacle, it can be thus hypothesized that silencing of the latter could facilitate the release of UBF from rDNA, while enhanced treacle expression may interfere with cisplatin-induced apoptosis. Consistently with this hypothesis, both silencing and ectopic expression of *TCOF1* affect the expression of crucial apoptotic regulators [86]; however, whether treacle indeed contributes to cisplatin resistance needs to be experimentally verified.

The possible treacle involvement in cancer is also suggested by its reliance on kinase-regulated signaling pathways. As mentioned above, treacle-governed regulation of ribosome modifications and global translation programs is largely dependent of CK2-mediated phosphorylation. Protein kinase CK2 is a well-known regulator of cancer development and progression, as well as an important target for anticancer therapies [99,100,101]. It can thus be expected that disturbed CK2 activity in cancer cells may affect the functioning of treacle, thereby contributing to cancer-specific translation programs.

All these data, although limited, suggest that *TCOF1* dysfunction may possibly contribute to cancer development and progression. However, clearly further studies are needed that aim at the analysis of *TCOF1* alterations in large cohorts of cancer patients. The promising results of studies indicating that *TCOF1* silencing sensitizes cancer cells to radiotherapy and cisplatin treatment (summarized in Figure S2) suggest that drugs targeting treacle could be possibly used in combination therapies. Interestingly, such therapeutic approach was recently proposed for an inhibitor of PFKFB3 enzyme that, similarly to treacle, interacts with MRN complex and enables recruitment of HR proteins and DNA repair [102]. The small inhibitor of PFKFB3 is currently under preclinical studies (https://kancera.com/en/researchportfolio/, accessed on 11 May 2020).

### 5.3. The other Pathologies Linked with TCOF1 Dysfunction

It was suggested that treacle loss could contribute to the development of Hirschsprung disease (HSCR), a congenital disorder caused by the absence of enteric ganglia. HSCR is a multigenic disease, characterized by various inheritance patterns and variable penetration [31]. The enteric ganglia are derived from neural cell crest progenitors. *TCOF1* haploinsufficiency in mice delays NCCs migration in the developing gut due to enhanced apoptosis of neuroepithelial progenitors and sensitizes *Pax*3+/− mice agangliosis in the colon [31]. However, to our knowledge, there are no studies indicating that *TCOF1* dysfunction may influence development of Hirschsprung disease in human patients, and therefore the clinical significance of these findings requires further analysis.

## 6. Conclusions

**Figure 7:**
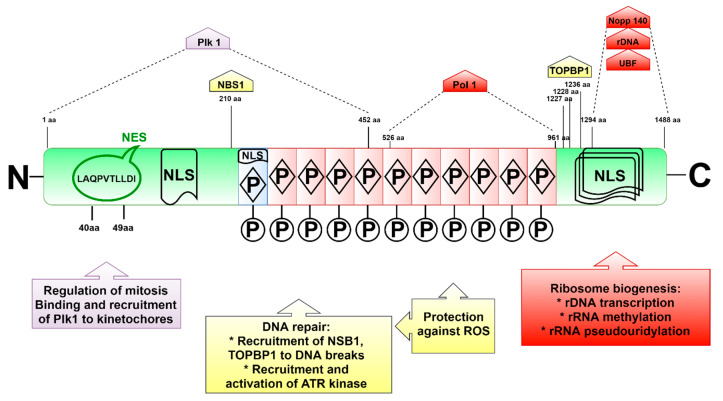
The summary of the key treacle functions in the cell. The molecules interacting with specific amino acids of Table 140. and UBF. In response to DNA damage, treacle binds and recruits NBS1 to the nucleolus, enabling silencing of rRNA transcription. Interaction with TOPBP1 enables recruitment and activation of ATR kinase, a crucial regulator of DNA damage response. Treacle function in the antioxidative defense is tightly linked with its role in DNA damage repair. The ability of treacle to regulate mitosis and cell cycle progression relies on its interaction with Plk1.

Treacle emerges as one of the crucial regulators of key cellular processes (Figure 7). It regulates ribosome biogenesis, mitosis, proliferation, and contributes to the cellular responses to DNA damage and apoptotic regulation. The role of *TCOF1* dysfunction in Treacher Collins syndrome is well documented. However, still, several important questions remain to be answered. Firstly, the mechanisms governing spatiotemporal regulation of *TCOF1* expression are largely unknown. The studies on transcriptional *TCOF1* regulators gave inconclusive results [18]. Since ribosome biogenesis can be regulated by non-coding RNAs [103], it may suggest that *TCOF1* could be a target of such regulation. Secondly, the specific mechanisms by which *TCOF1* mutations contribute to the TCS remain to be elucidated since the effects of ectopic expression of *TCOF1* mutants do not correspond with the molecular observations in cells of TCS patients. Ribosome biogenesis emerges as an attractive therapeutic target in oncology [104]. In accordance, the growing body of evidence suggests the involvement of treacle in cancer development and resistance to therapy. Future studies should focus on the systemic analysis of *TCOF1* alterations in cancer, its potential regulation by oncogenic signaling pathways, as well as its potential contribution to cancer progression and resistance to therapies. The nucleoli can control cellular functioning and homeostasis by acting as hubs that trap and immobilize proteins preventing their action in other cellular compartments [105,106]. It is possible that treacle can be involved in these “trapping” interactions. Finally, given the fact that treacle regulates the maturation of neural crest cells, which are the origin of multiple lineages, including bones, cartilages, neurons, glia, as well as endocrine cells, vascular smooth muscle cells, and melanocytes [107], it can be expected that *TCOF1* dysfunction may contribute to development of disorders related to at least some of these tissues and cell types.

## Data Availability

The data on *TCOF1* mutations in uterine carcinomas were retrieved from publicly available transcriptomic data of The Cancer Genome Atlas using cBio platform (http://www.cbioportal.org/, accessed on 11 May 2020).

## Supplementary Materials

The following are available online at https://www.mdpi.com/1422-0067/22/5/2482/s1: Figure S1: Transcripts encoding treacle protein, Figure S2: The role of treacle in response of cancer cells to DNA-damaging agents, Table S1: *TCOF1* mutations and SNPs identified in TCS patients.
