# Supplementary material for: The Role of TCOF1 Gene in Health and Disease: Beyond Treacher Collins Syndrome
Source: Int J Mol Sci. 2021 Mar 1;22(5):2482. doi: 10.3390/ijms22052482 (PMC7957619; doi:10.3390/ijms22052482)
Supplement: Supplementary file 1 [file ijms-22-02482-s001.zip › Sup/Table S1.docx]

**Table** S1. *TCOF1*mutations and SNPs identified in TCS patients. [1-3]

| **Location** | **cDNA** | **Amino acid change** | **Variant** | **Protein effect** | | **Clinical phenotype** | **Reference** |
| --- | --- | --- | --- | --- | --- | --- | --- |
| 5’-UTR | c.-89T>G |  | Polymorphism |  | | TCS | [4] |
| 5'-UTR | c.-59G>A |  | Unclassified variants |  | | TCS | [5] |
| 5’-UTR | c.-41G>T |  | Polymorphism |  | | TCS | [6] |
| 5’-UTR | c.-26T>A |  | Polymorphism |  | | TCS | [4] |
| exon 1 | c.1A>T | p.M1? | Mutation |  | | TCS | [5] |
| exon 1 | c.3G>A | p.M1? | Mutation |  | | TCS | [5,7] |
| exon 1 | c.40A>T | p.I14F | Mutation |  | | TCS | [8] |
| exon 1 | c.42C>G | p.I14M | Mutation |  | | TCS | [5] |
| exon 1 | c.50A>G | p.H17R | Mutation |  | | TCS | [5] |
| exon 1 | c.59delG |  | Mutation |  | | TCS | [8] |
| exon 1 | c.61G>C | p.A21P | Mutation |  | | TCS | [5] |
| exon 1 | c.77C>T | p.A26V | Mutation |  | | TCS | [9] |
| exon 1 | c.79G>A | p.A27T | Mutation |  | | TCS | [5] |
| exon 1 | c.87delA | p.V30* | Mutation | STOP | | TCS | [5] |
| exon 1 | ND | p.Q36* | Mutation | STOP | | TCS | [10] |
| intron 1 | c.108+62_68del |  | Unclassified variants |  | | TCS | [5] |
| intron 1 | c.109-1delG |  | Mutation |  | | TCS | [10] |
| intron 1 | c.109-2A>T |  | Mutation |  | | TCS | [5] |
| intron 1 | c.109-28T>C |  | Unclassified variants |  | | TCS | [5] |
| exon 2 | c.122C>T | p.A41V | Polymorphism |  | | TCS | [11] |
| exon 2 | c.149A>G | p.Y50C | Mutation |  | | TCS | [12] |
| exon 2 | c.157T>C | p.W53R | Mutation |  | | TCS | [10,13] |
| exon 2 | c.159G>A | p.W53* | Mutation | STOP | | TCS | [5] |
| exon 2 | c.163C>T | p.Q55* | Mutation | STOP | | TCS | [5,9] |
| intron 2 | 1244G>T |  | Polymorphism |  | | TCS | [4] |
| exon 3 | c.165‑1G>A |  | Mutation | STOP | | TCS | [14] |
| exon 3 | c.218insAACC | p.A73fs | Mutation |  | | TCS | [15] |
| exon 3 | c.274 delG |  | Mutation |  | | TCS | [10] |
| exon 3 | ND | p.E93* | Mutation |  | | TCS | [10] |
| exon 3 | c.303_304delCA | p.A101Afsx73 | Mutation | STOP | | TCS | [6] |
| exon 3 | c.4369_4373 | p.K1457-Efs*12 | ND | affects NLS | | TCS | [16] |
| intron 3 | c.304+5G>C | splice | Mutation |  | | TCS | [5,8,10] |
| intron 3 | c.305-1 G>A |  | Mutation |  | | TCS | [12] |
| intron 3 | c.305-52A>G |  | Polymorphism |  | | TCS | [5] |
| exon 4 | c.343delG | p.121* | Mutation | STOP | | TCS | [7] |
| exon 4 | c.357_358delAA | p.S120fs | Mutation |  | | TCS | [5] |
| exon4/intron4 | c.376delAAGGTGAGTGGGACTGCC |  | ND | STOP | | TCS | [17] |
| intron 4 | c.379-2A>G |  | Mutation |  | | TCS | [7] |
| exon 5 | c.381_382delAG | p.A127* | Mutation | STOP | | TCS | [18] |
| exon 5 | c.389insA |  | Mutation |  | | TCS | [8] |
| exon 5 | c.390delGA | p.173* | Mutation | STOP | | TCS | [7] |
| exon 5 | c.405_406delTG | p.G136fs | Mutation |  | | TCS | [5,10,19] |
| exon 5 | c.408delG | p.214* | Mutation |  | | TCS | [7] |
| exon 5 | c.422insA | p.H141Q | Mutation | STOP | | TCS | [20] |
| exon 5 | c.431delC | p.T144fs | Mutation |  | | TCS | [15] |
| exon 5 | c.475A>T | p.K159* | Mutation | STOP | | TCS | [9] |
| exon 5 | c.484_668ins |  | ND | STOP | | TCS | [21] |
| exon 5 | c.489delC | p.S164Qfs*55 | Mutation |  | | TCS | [22] |
| exon 5 | c.497delATAC | p.N166I | Mutation | STOP | | TCS | [20] |
| exon 5 | c.503C>T | p.T168M | Polymorphism |  | | TCS | [7] |
| exon 5 | c.519delT | p.T173Tfsx46 | Mutation | STOP | | TCS | [6] |
| intron 5 | c.565+1G>A |  | Mutation |  | | TCS | [5] |
| intron 5 | c.565+18G>C |  | Polymorphism |  | | TCS | [7] |
| intron 5 | c.566-10C>A |  | Mutation |  | | TCS | [5] |
| exon 6 | c.574del(16bp) | p.209* | Mutation | STOP | | TCS | [7] |
| exon 6 | c.579G>A | p.A193A | Unclassified variants |  | | TCS | [5] |
| exon 6 | c.599delG | p.S200Tfsx19 | Mutation | STOP | | TCS | [6] |
| exon 6 | c.618delC | p.S207fs | Mutation |  | | TCS | [5] |
| exon 6 | c.630 delAG |  | Mutation |  | | TCS | [10] |
| intron 6 | c.639+1G>A |  | Mutation |  | | TCS | [15] |
| intron 6 | c.639+32C>G |  | Polymorphism |  | | TCS | [6,23] |
| intron 6 | c.639+32C>T¹ |  | Polymorphism |  | | TCS | [7,23] |
| intron 6 | c.640-69T>C |  | Polymorphism |  | | TCS | [5] |
| intron 6 | c.14036G>A |  | Polymorphism |  | | TCS | [4] |
| exon 6A | c.648delC | p.S217Qfs*2 | Mutation |  | | TCS | [22] |
| exon 6A | c.726delT | p.K244fs | Mutation |  | | TCS | [13] |
| exon 6A | c.827_844del | p.G276_E281del | Mutation |  | | TCS | [5] |
| exon 7 | IVS6-710 C-T |  | Mutation |  | | TCS | [24] |
| exon 7 | c.698delC |  | Mutation |  | | TCS | [8] |
| exon 7 | c.720-727delAGCACCCC |  | Mutation |  | | TCS | [12,24] |
| exon 7 | c.724insC |  | Mutation |  | | TCS | [10] |
| exon 7 | c.728insC | p.271* | Mutation | STOP | | TCS | [7] |
| exon 7 | c.744insT |  | Mutation | STOP | | TCS | [25] |
| exon 7 | ND | p.Q252* | Mutation |  | | TCS | [10] |
| exon 7 | c.768 G>A | p.G256G | Polymorphism |  | | TCS | [7] |
| exon 7 | c.786delA |  | Mutation |  | | TCS | [12] |
| exon 7 | c.786delAG |  | Mutation | STOP | | TCS | [25] |
| exon 7 | c.790_791delAG | p.S264QfsX7 | Mutation |  | | TCS | [26] |
| exon 7 | c.797G>A | p.S266N | Mutation |  | | TCS | [7] |
| exon 7 | c.803A>G¹ | p.E268G | Polymorphism |  | | TCS | [23] |
| exon 7 | c.1016C>G | p.S339* | Mutation | STOP | | TCS | [5] |
| exon 7 | c.1028G>A | p.S343N | Unclassified variants |  | | TCS | [5] |
| exon 7 | c.1347T>C | p.P439L | Polymorphism |  | | TCS | [6,20] |
| intron 7 | c.852+39A>G |  | Polymorphism |  | | TCS | [7] |
| intron 7 | c.1083+39G>A |  | Polymorphism |  | | TCS | [5] |
| exon 8 | c.864-865delAG |  | Mutation |  | | TCS | [8] |
| exon 8 | c.911C>T | p.S304L | Mutation |  | | TCS | [27] |
| exon 8 | c.998 C>T¹ | p.S333L | Polymorphism | affects ATM phoshorylation site | | TCS | [23] |
| exon 8 | c.1015 G>T |  | Mutation |  | | TCS | [12] |
| exon 8 | c.1086G>A | p.A362A | Unclassified variants |  | | TCS | [5] |
| exon 8 | c.1095_1096delAG | p.G366fs | Mutation |  | | TCS | [8,15] |
| exon 8 | c.1096G>T | p.G366* | Mutation | STOP | | TCS | [5] |
| exon 8 | c.1242C>T | p.S414S | Unclassified variants |  | | TCS | [5] |
| intron 8 | c.1047+60 G>C |  | Polymorphism |  | | TCS | [7] |
| intron 8 | c.1278+60G>C |  | Polymorphism |  | | TCS | [5] |
| exon 9 | c.1084G>A | p.A362T | Mutation |  | | TCS | [28] |
| exon 9 | c.1098delC |  | Mutation |  | | TCS | [12] |
| exon 9 | c.1099A>T | p.K367* | Mutation | STOP | | TCS | [7] |
| exon 9 | c.1106insC |  | Mutation | affects ATM phoshorylation site | | TCS | [8] |
| exon 9 | c.1120delG |  | Mutation |  | | TCS | [10] |
| exon 9 | c.1142delC |  | Mutation |  | | TCS | [29] |
| exon 9 | c.1150G>A, 3987insG | p.A350A | Mutation |  | | TCS | [23] |
| exon 9 | c.1164insT |  | Mutation |  | | TCS | [23] |
| exon 9 | c.1215insA |  | Mutation |  | | TCS | [12] |
| exon 9 | c.1216insA |  | Mutation |  | | TCS | [10] |
| exon 9 | c.1230A>G | p.E410E | Polymorphism |  | | TCS | [7] |
| exon 9 | c.1247A >G | p.E419E | Polymorphism |  | | TCS | [28] |
| exon 9 | c.1281G>A | p.A427A | Polymorphism |  | | TCS | [5] |
| exon 9 | c.1298delC | p.A433fs | Mutation |  | | TCS | [15] |
| exon 9 | c.1303delC | p.Q435fs | Mutation |  | | TCS | [5] |
| exon 9 | c.1446dupA | p.D483fs | Mutation |  | | TCS | [5,12,19] |
| exon 9 | c.1473dupC | p.M492fs | Mutation |  | | TCS | [5] |
| exon 10 | c.1287insG |  | Mutation |  | | TCS | [23] |
| exon 10 | c.1316C>T | p.P439L | Polymorphism |  | | TCS | [10,20,23] |
| exon 10 | c.1327insA |  | Mutation |  | | TCS | [10] |
| exon 10 | c.1347T>C¹ | p.P449P | Mutation/Polymorphism |  | | TCS | [7,20,23,24,28,30] |
| exon 10 | c.1359G>A | p.G453G | Mutation |  | | TCS | [28] |
| exon 10 | c.1393 C>T | p.Q465* | Mutation | affects NLS | | TCS | [29] |
| exon 10 | c.1406-1409delAGAG |  | Mutation |  | | TCS | [23,24] |
| exon 10 | c.1408-1409delAG | p.S470Q | Mutation | STOP | | TCS | [12,20,23,24] |
| exon 10 | c.1441insGG |  | Mutation |  | | TCS | [10] |
| exon 10 | c.1557dupA | p.G520fs | Mutation |  | | TCS | [5,31] |
| exon 10 | c.1578 T > C | p.P526P | Mutation/Polymorphism | Affects PolI interaction site | | TCS | [6,13,20] |
| exon 10 | c.1581delG | p.G587Gfsx69 | Mutation | STOP/Affects PolI interaction site | | TCS | [6] |
| exon 10 | c.1609C>T | p.Q537* | Mutation | STOP/Affects PolI interaction site | | TCS | [15] |
| exon 10 | c.1639_1640delAG | p.S547fs | Mutation | Affects PolI interaction site | | TCS | [5,6,12,20,32] |
| exon 10 | c.1702C>T | p.Q568* | Mutation | STOP/Affects PolI interaction site | | TCS | [5] |
| intron 10 | c.1473+68C>T |  | Polymorphism |  | | TCS | [7] |
| exon 11 | c.1530G>T | p.G510G | Polymorphism | Affects PolI interaction site | | TCS | [7,23,28,30] |
| exon 11 | c.1552G>A | p.V518I | Polymorphism | Affects PolI interaction site | | TCS | [7,20,23] |
| exon 11 | c.[1552delG; 1565T< | p.518* | Mutation | STOP/Affects PolI interaction site | | TCS | [7] |
| exon 11 | c.1611G>A | p.S537S | Mutation/Polymorphism | Affects PolI interaction site | | TCS | [7,20,23,24,28,30] |
| exon 11 | c.1729C>T | p.Q577* | Mutation | STOP/Affects PolI interaction site | | TCS | [5] |
| exon 11 | c.1747C>T |  | Mutation | STOP/Affects PolI interaction site | | TCS | [33] |
| exon 11 | c.1761G>T | p.G587G | Polymorphism | Affects PolI interaction site | | TCS | [6,23] |
| exon 11 | c.1762C>G¹ | p.P588A | Mutation | Affects PolI interaction site | | TCS | [30] |
| exon 11 | c.1782_1788del7 | p.Val595fs | Mutation | Affects PolI interaction site | | TCS | [5] |
| exon 11 | c.1813_1814delAT | p.M605fs | Mutation | Affects PolI interaction site | | TCS | [5] |
| exon 11 | c.1837delG | p.E613fs | Mutation | Affects PolI interaction site | | TCS | [5] |
| exon 11 | c.1842A>G | p.S614S | Polymorphism | Affects PolI interaction site | | TCS | [6,20] |
| exon 11 | c.1854G>A | p.A618A | Unclassified variants | Affects PolI interaction site | | TCS | [5] |
| exon 11 | c.17681C>T | p.P526P | Polymorphism | Affects PolI interaction site | | TCS | [4] |
| exon 11 | c.17693G>A | p.G530G | Polymorphism | Affects PolI interaction site | | TCS | [4] |
| exon 12 | c.1687C>T | p.Q563* | Mutation | STOP/Affects PolI interaction site | | TCS | [7] |
| exon 12 | c.1719delG | p.N574Tfs | Mutation | Affects PolI interaction site | | TCS | [29] |
| exon 12 | c.1742insC | p.620* | Mutation | STOP/Affects PolI interaction site | | TCS | [7] |
| exon 12 | c.1762C>G | p.P588A | Polymorphism | Affects PolI interaction site | | TCS | [7,28] |
| exon 12 | c.1768insC | p.620* | Mutation | STOP/Affects PolI interaction site | | TCS | [7] |
| exon 12 | c.1837G > C | p.A588P | Polymorphism | Affects PolI interaction site | | TCS | [6] |
| exon12 | c.1863A>G, 2355-2364delCAGGGCCAGA | p.E621E | Mutation | Affects PolI interaction site | | TCS | [23] |
| exon 12 | c.1866-1873delAGATAGTG |  | Mutation | Affects PolI interaction site | | TCS | [23] |
| exon 12 | c.1867-1868delGA | p.D623X | Mutation | STOP/Affects PolI interaction site | | TCS | [9,23] |
| exon 12 | c.1868delATAG | p.632* | Mutation | STOP/Affects PolI interaction site | | TCS | [7] |
| exon 12 | c.1872-1875delTGAG |  | Mutation | Affects PolI interaction site | | TCS | [12] |
| exon 12 | c.1879 delGAGAA |  | Mutation | Affects PolI interaction site | | TCS | [10] |
| exon 12 | c.1907delT | p.L636fs | Mutation | Affects PolI interaction site | | TCS | [5] |
| exon 12 | c.1952_1956delCTGCA | p.T651fs | Mutation | Affects PolI interaction site | | TCS | [5] |
| exon 12 | c.1953C>T | p.T651T | Unclassified variants | Affects PolI interaction site | | TCS | [5] |
| exon 12 | c.1973delC | p.P658Lfsx53 | Mutation | STOP/Affects PolI interaction site | | TCS | [6] |
| exon 12 | c.1993C>G | p.A665P | Polymorphism | Affects PolI interaction site | | TCS | [6] |
| exon 12 | c.1993delG | p.A665fs | Mutation | Affects PolI interaction site | | TCS | [5] |
| exon 12 | c.1999dupC | p.R667fs | Mutation | Affects PolI interaction site | | TCS | [5,7] |
| exon 12 | c.2065_2075del11 | p.P689fs | Mutation | Affects PolI interaction site | | TCS | [5] |
| exon 12 | c.2098_2099delGA | p.D700* | Mutation | STOP/Affects PolI interaction site | | TCS | [5,23] |
| exon 12 | c.2099_2102delATAG | p.D700fs | Mutation | Affects PolI interaction site | | TCS | [5,7] |
| exon 12 | c.2103_2106delTGAG | p.S701fs | Mutation | Affects PolI interaction site | | TCS | [5,12] |
| exon 12 | c.18111A>G | p.S614S | Polymorphism | Affects PolI interaction site | | TCS | [4] |
| intron 12 | c.1911+1G>A |  | Mutation |  | | TCS | [7] |
| intron 12 | c.1911+36delC |  | Polymorphism |  | | TCS | [7] |
| intron 12 | c.2142+22C>T |  | Unclassified variants |  | | TCS | [5] |
| exon 13 | c.1611G>A |  | Mutation | Affects PolI interaction site | | TCS | [24] |
| exon 13 | c.18434G>C | p.A665P | Polymorphism | Affects PolI interaction site | | TCS | [4] |
| exon 13 | c.1915-1916delAA |  | Mutation | Affects PolI interaction site | | TCS | [8] |
| exon 13 | c.1926-1927insG |  | Mutation | Affects PolI interaction site | | TCS | [12] |
| exon 13 | c.1974G>C¹ | p.L658F | Polymorphism | Affects PolI interaction site | | TCS | [23] |
| exon 13 | c.1978delC |  | Mutation | STOP/Affects PolI interaction site | | TCS | [21] |
| exon 13 | c.2014insG |  | Mutation | Affects PolI interaction site | | TCS | [10] |
| exon 13 | c.2014C>T¹ | p.P672S | Polymorphism | Affects PolI interaction site | | TCS | [23] |
| exon 13 | c.2018-2025delCAGTCACC |  | Mutation | Affects PolI interaction site | | TCS | [23,24] |
| exon 13 | c.2019-2025delAGTCACC |  | Mutation | Affects PolI interaction site | | TCS | [8] |
| exon 13 | c.2026C>T | p.Q676* | Mutation | STOP; affects ATM phosphorylation site, Affects PolI interaction site | | TCS | [8,12] |
| exon 13 | c.2055delAG |  | Mutation | STOP/Affects PolI interaction site | | TCS, AMD? | [34] |
| exon 13 | c.2059delAG |  | Mutation | STOP; Affects PolI interaction site | | TCS | [25] |
| exon 13 | c.2082-2085delTGAG |  | Mutation | Affects PolI interaction site | | TCS | [8] |
| exon 13 | c.2157dupG | p.K720fs | Mutation | Affects PolI interaction site | | TCS | [5,12] |
| exon 13 | c.2164delC | p.L722fs | Mutation | Affects PolI interaction site | | TCS | [5] |
| exon 13 | c.2167C>T | p.Q723* | Mutation | STOP/Affects PolI interaction site | | TCS | [5] |
| exon 13 | c.2285_2286delCT | p.S762* | Mutation | STOP/Affects PolI interaction site | | TCS | [5] |
| exon 13 | c.2285_2286delCT | p.S762fs | Mutation | STOP/Affects PolI interaction site | | TCS | [6] |
| exon 13 | c.2287G>T | p.E763* | Mutation | STOP/Affects PolI interaction site | | TCS | [5] |
| intron 13 | c.2341-2A>G |  | Mutation |  | | TCS | [5] |
| exon 14 | c.2110delG |  | Mutation | Affects PolI interaction site | | TCS | [8] |
| exon 14 | c.2221C> T | p.Q741* | Mutation | STOP/Affects PolI interaction site | | TCS | [28] |
| exon 14 | ND | p.K748K | Mutation | Affects PolI interaction site | | TCS | [19,23] |
| exon 14 | ND | p.K749K | Mutation | Affects PolI interaction site | | TCS | [10] |
| exon 14 | c.2205insTT; 2206delG | p.795* | Mutation | STOP/Affects PolI interaction site | | TCS | [7] |
| exon 14 | c.2426delC | p.P809fs | Mutation | Affects PolI interaction site | | TCS | [5] |
| exon 14 | c.2473_2476delTCCA | p.S825fs | Mutation | Affects PolI interaction site | | TCS | [5] |
| exon 14 | c.2478G>A |  | Mutation | Affects PolI interaction site | | TCS | [13,15,19,23] |
| intron 14 | c.2247+27G>A |  | Polymorphism |  | | TCS | [7] |
| intron 14 | c.2248-42insG |  | Polymorphism |  | | TCS | [7] |
| intron 14 | c.2478+5G>C |  | Mutation |  | | TCS | [5] |
| intron 14 | c.2478+5G>A |  | Mutation |  | | TCS | [22] |
| exon 15 | c.2272C>T | p.Q758* | Mutation | STOP/Affects PolI interaction site | | TCS | [8] |
| exon 15 | c.2297delG |  | Mutation | Affects PolI interaction site | | TCS | [10] |
| exon 15 | c.2354-2363delCAGGGCCAGA |  | Mutation | Affects PolI interaction site | | TCS | [23] |
| exon 15 | c.2355-2356delAG |  | Mutation | Affects PolI interaction site | | TCS | [23] |
| exon 15 | c.2375-2376delGG |  | Mutation | Affects PolI interaction site | | TCS | [12] |
| exon 15 | c.238delG |  | Mutation | Affects PolI interaction site | | TCS | [10] |
| exon 15 | c.2394_2395delAG | p.D799Qfs* | Mutation | STOP | | TCS | [18] |
| exon 15 | c.2399delGTGA |  | Mutation | Affects PolI interaction site | | TCS | [10] |
| exon 15 | c.2399-2402delGTGA |  | Mutation | Affects PolI interaction site | | TCS | [8] |
| exon 15 | ND | p.E796* | Mutation | STOP/Affects PolI interaction site | | TCS | [23] |
| exon 15 | c.2626_2627delGA | p.D876Qfsx2 | Mutation | STOP/Affects PolI interaction site | | TCS | [6] |
| intron 15 | c.2428-20insCTCT |  | Polymorphism |  | | TCS | [20,23] |
| intron 15 | c.2428-20insCTCTC |  | Polymorphism |  | | TCS | [7,20] |
| intron 15 | c.2428-36A>C |  | Polymorphism |  | | TCS | [7] |
| intron 15 | c.2659-28delTCTC |  | Polymorphism |  | | TCS | [6] |
| exon 16 | c.2429C>T¹ | p.A810V | Mutation/Polymorphism | Affects PolI interaction site | | TCS | [7,10,20,23,28,30] |
| exon 16 | c.2442delG |  | Mutation | Affects PolI interaction site | | TCS | [10] |
| exon 16 | c.2452C>T | p.Q818* | Mutation | STOP/Affects PolI interaction site | | TCS | [7] |
| exon 16 | c.2490delC | p.PR830–831PG² | Mutation | STOP/Affects PolI interaction site | | TCS | [20] |
| exon 16 | c.2526insA | p.854* | Mutation | STOP/Affects PolI interaction site | | TCS | [7] |
| exon 16 | c.2526delAG | p.TG842–843TA² | Mutation | STOP/Affects PolI interaction site | | TCS | [20] |
| exon 16 | c.2527insA |  | Mutation | Affects PolI interaction site | | TCS | [23] |
| exon 16 | c.2529ins G |  | Mutation | STOP/Affects PolI interaction site | | TCS | [25] |
| exon 16 | c.2534C>T¹ | p.S845L | Polymorphism | Affects PolI interaction site | | TCS | [20,23] |
| exon 16 | c.2542C>T | p.G848* | Mutation | STOP/Affects PolI interaction site | | TCS | [7] |
| exon 16 | c.2545delG | p.872* | Mutation | STOP/Affects PolI interaction site | | TCS | [7] |
| exon 16 | c.2552delA; 2561delA | p.K851S | Mutation | STOP/Affects PolI interaction site | | TCS | [20] |
| exon 16 | c.2565delAG | p.SG855–856SE² | Mutation | STOP/Affects PolI interaction site | | TCS | [20] |
| exon 16 | c.2622insT |  | Mutation | Affects PolI interaction site | | TCS | [8] |
| exon 16 | c.2660C>T | p.A887V | Polymorphism | Affects PolI interaction site | | TCS | [6,20] |
| exon 16 | c.2683C>T | p.Q895* | Mutation | STOP/Affects PolI interaction site | | TCS | [5,7] |
| exon 16 | c.2713G>T | p.E905* | Mutation | STOP/Affects PolI interaction site | | TCS | [5] |
| exon 16 | c.2762C>T | p.P921L | Unclassified variants | Affects PolI interaction site | | TCS | [5] |
| exon 16 | c.2765C>T | p.S992L | Polymorphism | Affects PolI interaction site | | TCS | [6,20] |
| exon 16 | c.2831delA | p.E944Efsx6 | Mutation | Affects PolI interaction site | | TCS | [6] |
| intron 16 | c.2628+26A>G |  | Polymorphism |  | | TCS | [7] |
| intron 16 | c.2629-1G>A |  | Mutation |  | | TCS | [7] |
| intron 16 | c.2629-1G>C |  | Mutation |  | | TCS | [12] |
| intron 16 | c.2629-3A>G |  | Mutation |  | | TCS | [7] |
| intron 16 | c.2859+26A>G |  | Unclassified variants |  | | TCS | [5] |
| intron 16 | c.2859-30G>A |  | Polymorphism |  | | TCS | [6] |
| intron 16 | c.2860-27G>A |  | Polymorphism |  | | TCS | [5] |
| intron 16 | c.21761-21765delCTCTC |  | Polymorphism |  | | TCS | [4] |
| exon 16A | IVS16a +C/T |  | ND |  | | TCS | [30] |
| exon 16A | c.2919G>T | p.R973S | Unclassified variants |  | | TCS | [5] |
| intron 16A | exon 16A+4C>T |  | Polymorphism |  | | TCS | [7] |
| intron 16A | c.2859+3444C > T |  | Polymorphism |  | | TCS | [6] |
| exon 17 | c.2629G>A | p.V877M | Mutation |  | | TCS | [7] |
| exon 17 | c.2643C>T | p.P881P | Polymorphism |  | | TCS | [7] |
| exon 17 | c.2643C>T | p.P881P | Polymorphism |  | | TCS | [28] |
| exon 17 | c.2731C>T | p.R911* | Mutation | STOP | | TCS | [25,33] |
| exon 17 | c.2740G>T | p.S914* | Mutation | STOP | | TCS | [8] |
| exon 17 | c.2800C>T | p.Q934* | Mutation | STOP; affects ATM phosphorylation site | | TCS | [7] |
| exon 17 | c.2802A>G | p.Q934Q | Polymorphism |  | | TCS | [28] |
| exon 17 | c.2924C>T | p.P975L | Polymorphism |  | | TCS | [6] |
| exon 17 | c.2962C>T | p.R988* | Mutation | STOP | | TCS | [5] |
| exon 17 | c.2969C>A | p.S990* | Mutation | STOP | | TCS | [5] |
| exon 17 | c.21786T>C | p.V887A | Polymorphism |  | | TCS | [4] |
| exon 17 | c.21968G>T | p.A948S | Polymorphism |  | | TCS | [4] |
| intron 17 | c.2815+1delG |  | Mutation |  | | TCS | [12] |
| intron 17 | c.3046+1G>A |  | Mutation |  | | TCS | [5] |
| intron 17 | c.3047-1G>A |  | Mutation |  | | TCS | [5] |
| intron 17 | c.3047-2A>T |  | Mutation |  | | TCS | [5] |
| intron 17 | c.3047-2A>G |  | Mutation |  | | TCS | [22] |
| exon 18 | c.2822-2823delGA |  | Mutation |  | | TCS | [23] |
| exon 18 | c.2846delC |  | Mutation |  | | TCS | [12] |
| exon 18 | c.2881delG |  | Mutation |  | | TCS | [12] |
| exon 18 | c.2952G>T | p.Q984H | Mutation | affects ATM phoshorylation site | | TCS | [25] |
| exon 18 | c.3053_3054delGA | p.R1018fs | Mutation |  | | TCS | [15,23] |
| exon 18 | c.3112delG | p.A1038fs | Mutation |  | | TCS | [5,12] |
| exon 18 | c.3118_3119dupG | p.A1040Gfx47 | Mutation |  | | TCS | [6] |
| exon 18 | c.3156C>T |  | Mutation |  | | TCS | [5] |
| exon 18 | c.3163C>T | p.Q1055* | Mutation | STOP | | TCS | [5] |
| exon 18 | c.3183>4A |  | Mutation |  | | TCS | [5] |
| exon 19 | c.2998delC |  | Mutation |  | | TCS | [10] |
| exon 19 | c.3066+59C>T, 2355delCAGGGCCAGA |  | Mutation |  | | TCS | [23] |
| exon 19 | c.3156C>T | p.G1052G | ND | STOP | | TCS | [35] |
| intron 19 | c.3066+66C>T¹ |  | Polymorphism |  | | TCS | [7,23] |
| intron 19 | c.3067-72A>C |  | Polymorphism |  | | TCS | [7] |
| intron 19 | c.3197+66C>T |  | Polymorphism |  | | TCS | [6] |
| exon 20 | c.3084delA |  | Mutation |  | | TCS | [12] |
| exon 20 | c.3086-3092delCACTCCC |  | Mutation |  | | TCS | [23] |
| exon 20 | c.3100delA |  | Mutation |  | | TCS | [23] |
| exon 20 | ND | p.Q1041* | Mutation | STOP | | TCS | [10] |
| exon 20 | c.3169C>T | p.Q1057* | Mutation | STOP | | TCS | [7] |
| exon 20 | c.3279C>T¹ | p.H1093H | Polymorphism |  | | TCS | [23] |
| exon 20 | c.3314delG | p.G1105fs | Mutation |  | | TCS | [5] |
| exon 20 | c.3456_63delTTCTTCAG | p.S1152Rfsx3 | Mutation | STOP | | TCS | [6] |
| intron 20 | c.3287-34G>A |  | Polymorphism |  | | TCS | [7] |
| intron 20 | c.3517-34G>A |  | Polymorphism |  | | TCS | [6] |
| exon 21 | c.2103_2106delTGAG | p.S701fs | Mutation |  | | TCS | [12,15] |
| exon 21 | c.3276G>C | p.R1099P | Polymorphism |  | | TCS | [28] |
| exon 21 | c.3296C>G | p.P1099R | Polymorphism |  | | TCS | [7,23] |
| exon 21 | c.3311delC |  | Mutation | Affects TOPBP1 interaction site | | TCS | [23] |
| exon 21 | c.3527C>G | p.P1176R | Polymorphism |  | | TCS | [6,23] |
| exon 21 | c.3528C>T | p.P1176P | Unclassified variants |  | | TCS | [5] |
| intron 21 | IVS21-3C/T |  | ND |  | | TCS | [30] |
| intron 21 | c.3600+1delG |  | Mutation | Affects TOPBP1 interaction site | | TCS | [5] |
| intron 21 | c.3600+64dupT |  | Unclassified variants |  | | TCS | [5] |
| intron 21 | c.3370-3C>T |  | Polymorphism |  | | TCS | [6,7,12] |
| exon 22 | c.3376-3380delCTCTC |  | Mutation |  | | TCS | [8] |
| exon 22 | c.3389T>A | p.M1130K | Mutation |  | | TCS | [12] |
| exon 22 | c.3447T>C | p.D1149D | Polymorphism |  | | TCS | [28] |
| exon 22 | c.3498T>C | p.*1168Q | Polymorphism |  | | TCS | [28] |
| exon 22 | c.3611C>G | p.S1204* | Mutation | STOP | | TCS | [5] |
| exon 22 | c.3612A>C |  | Mutation | loss of exon 22 | | TCS | [11] |
| exon 22 | c.3613G>A |  | Mutation |  | | TCS | [5] |
| exon 22 | c.3700_3704delACTCT | p.T1234Gfsx5 | Mutation |  | | TCS | [6] |
| exon 22 | c.3745_3747delinsA | p.P1249fs | Mutation |  | | TCS | [5] |
| intron 22 | c.3550+1G-A |  | Mutation |  | | TCS | [10] |
| intron 22 | c.3781+2dupT |  | Mutation |  | | TCS | [5] |
| intron 22 | c.3781+8A>G |  | Unclassified variants |  | | TCS | [5] |
| exon 23 | c.3606delAG |  | Mutation |  | | TCS | [10] |
| exon 23 | c.3639delG |  | Mutation |  | | TCS | [12,24] |
| exon 23 | c.3700delG |  | Mutation |  | | TCS | [8] |
| exon 23 | c.3711-3712delAG |  | Mutation |  | | TCS | [23] |
| exon 23 | c.3779insC |  | Mutation |  | | TCS | [12] |
| exon 23 | c.3789G>A | p.K1263K | Mutation |  | | TCS | [28] |
| exon 23 | c.3802C>A¹ | p.R1268S | Polymorphism |  | | TCS | [23] |
| exon 23 | c.3819G>A¹ | p.S1273S | Polymorphism |  | | TCS | [23] |
| exon 23 | c.3823delC | p.R1275fs | Mutation |  | | TCS | [5] |
| exon 23 | c.3830delC |  | Mutation |  | | TCS | [12] |
| exon 23 | c.3853delC | p.Q1285fs | Mutation |  | | TCS | [15] |
| exon 23 | c.3853dupC | p.Q1285fs | Mutation |  | | TCS | [5,15] |
| exon 23 | c.3876 delCGGGGAAGGTGGGGAGGCCTCTGTT and insTTC |  | Mutation | STOP | | TCS | [25] |
| exon 23 | c.3899delT |  | Mutation |  | | TCS | [12] |
| exon 23 | c.3933insG |  | Mutation |  | | TCS | [12,24] |
| exon 23 | c.3938C>T¹ | p.A1313V | Mutation/Polymorphism |  | | TCS | [7,10,20,23,24] |
| exon 23 | c.3938T>C | p.V1313A | Polymorphism |  | | TCS | [28] |
| exon 23 | c.3942A>C | p.S1314S | Unclassified variants |  | | TCS | [5] |
| exon 23 | c.3975-3979delGAAAG |  | Mutation |  | | TCS | [12] |
| exon 23 | c.3983_3986delAGAA | p.K1328fs | Mutation |  | | TCS | [5] |
| exon 23 | c.3987insG | p.1352* | Mutation | STOP | | TCS | [7,12,23] |
| exon 23 | c.4019-4032delCAGAAGAGGAGCTT |  | Mutation |  | | TCS | [12] |
| exon 23 | c.4061G>C¹ | p.G1354A | Polymorphism |  | | TCS | [7,23] |
| exon 23 | c.4061delC | p.P1354fs | Mutation |  | | TCS | [12,15] |
| exon 23 | c.4108delAA |  | Mutation |  | | TCS | [10] |
| exon 23 | c.4111+5G>C |  | Mutation |  | | TCS | [29] |
| exon 23 | c.4127_4128delCT | p.S1376fs | Mutation |  | | TCS | [5] |
| exon 23 | c.4131_4132delTT | p.S1378fs | Mutation |  | | TCS | [5] |
| exon 23 | c.4177_4178delGA | p.D1393fs | Mutation | Affects NLS | | TCS | [5] |
| exon 23 | c.4218dupG | p.S1407fs | Mutation |  | | TCS | [5,7,12,13,15,23] |
| exon 23 | c.4219delT | p.S1407fs | Mutation |  | | TCS | [5] |
| exon 23 | c.4231C>T | p.Q1411* | Mutation | STOP; affects ATM phosphorylation site | | TCS | [3,5] |
| exon 23 | c.4295_4296AT>GA | p.D1432G | Unclassified variants |  | | TCS | [5] |
| exon 23 | c.4321delG | p.E1441fs | Mutation | Affects NLS | | TCS | [5] |
| exon 23 | c.4339_4340delAA | p.K1447fs | Mutation | Affects NLS | | TCS | [5,10] |
| exon 23 | c.4342A>T | p.R1448* | Mutation | STOP; affects NLS | | TCS | [5] |
| intron 23 | c.4112-17T>A |  | Polymorphism |  | | TCS | [7] |
| exon 23B | c.4169C>T | p.A1390V | Polymorphism |  | | TCS | [6,23] |
| exon 23B | c.4292G>C | p.G1431A | Polymorphism |  | | TCS | [6,23] |
| exon 23B | c.4331C>T | p.Q1411* | Mutation | STOP; affects ATM phosphorylation site | | TCS | [3,6] |
| exon 24 | IVS24+350C/G |  | Mutation |  | | TCS | [24] |
| exon 24 | c.4130delAAAAA |  | Mutation |  | | TCS | [10] |
| exon 24 | c.4130-4134delAAAAA |  | Mutation |  | | TCS | [12] |
| exon 24 | c.4131_4135delAAAAG | p.K1380Efs* | Mutation | STOP | | TCS | [18] |
| exon 24 | c.4131-4139delAAAAGAAAA | p.1378K-1380Kdel | Mutation |  | | TCS | [9] |
| exon 24 | c.4122delCA | p.1392* | Mutation | STOP; affects NLS | | TCS | [7] |
| exon 24 | c.4124-4125delAA |  | Mutation |  | | TCS | [12] |
| exon 24 | c.4134delA |  | Mutation |  | | TCS | [12] |
| exon 24 | c.4135 del GAAAA |  | Mutation | STOP | | TCS | [8,10,12,23-25] |
| exon 24 | c.4135-4136insG |  | Mutation |  | | TCS | [12] |
| exon 24 | c.4138_4142del | p.K1380 | Mutation | STOP | | TCS | [36] |
| exon 24 | c.4148insGAA | p.insK1382 | Polymorphism |  | | TCS | [7] |
| exon 24 | c.4156-4157delAA |  | Mutation |  | | TCS | [12] |
| exon 24 | c.4344dupA | p.R1448fs | Mutation | Affects NLS | | TCS | [15] |
| exon 24 | c.4355_4356ins14 | p.1456Thrfs*18 | Mutation | STOP | | TCS | [37] |
| exon 24 | c.4359_4363delAAAAA | p.E1453Efsx16 | Mutation |  | | TCS | [6,10] |
| exon 24 | c.4361_4365delAAAAA | p.K1454fs | Mutation | Affects NLS | | TCS | [10,12,15] |
| exon 24 | c.4362_4365delAAAA | p.E1456fs | Mutation | Affects NLS | | TCS | [5] |
| exon 24 | c.4363_4365delinsTAG | p.K1455* | Mutation | STOP; affects NLS | | TCS | [5] |
| exon 24 | c.4365delA | p.E1456fs | Mutation | Affects NLS | | TCS | [5,12] |
| exon 24 | c.4366_4369delGAAA | p.E1456fs | Mutation | Affects NLS | | TCS | [5] |
| exon 24 | c.4366_4370delGAAAA | p.K1457fs | Mutation | Affects NLS | | TCS | [6,10,12,13,23] |
| exon 24 | c.4369_4373delAAGAA | p.K1457fs | Mutation | Affects NLS | | TCS | [5,8,10,12,23,25,37] |
| exon 24 | c.4375_4377delAAG | p.K1459del | Mutation | Affects NLS | | TCS | [15] |
| exon 24 | c.4377_4379delGAA | p.K1460del | Unclassified variants | Affects NLS | | TCS | [5] |
| exon 24 | c.38922C>T | p.A1390V | Polymorphism |  | | TCS | [4] |
| intron 24 | IVS24+350C>G |  | Mutation |  | | TCS | [24] |
| intron 24 | IVS24+439C>A |  | Mutation |  | | TCS | [24] |
| intron 24 | c.4209+42C>A |  | Polymorphism |  | | TCS | [7] |
| intron 24 | c.4440+106G>T |  | Unclassified variants |  | | TCS | [5] |
| intron 24 | c.4440+108C>A |  | Polymorphism |  | | TCS | [5] |
| exon 25 | c.4224 G>A, 2822-2823delGA | p.E1508E | Mutation |  | | TCS | [23] |
| ND | c.2757_2758delAG | p.G920fs | Mutation |  | | TCS | [13,20] |
| **Location** | **cDNA** | **Amino acid change** | **Variant** | **Protein effect** | **Clinical phenotype** | | **Reference** |
| 5’-UTR | c.-89T>G |  | Polymorphism |  | TCS | | [4] |
| 5'-UTR | c.-59G>A |  | Unclassified variants |  | TCS | | [5] |
| 5’-UTR | c.-41G>T |  | Polymorphism |  | TCS | | [6] |
| 5’-UTR | c.-26T>A |  | Polymorphism |  | TCS | | [4] |
| exon 1 | c.1A>T | p.M1? | Mutation |  | TCS | | [5] |
| exon 1 | c.3G>A | p.M1? | Mutation |  | TCS | | [5,7] |
| exon 1 | c.40A>T | p.I14F | Mutation |  | TCS | | [8] |
| exon 1 | c.42C>G | p.I14M | Mutation |  | TCS | | [5] |
| exon 1 | c.50A>G | p.H17R | Mutation |  | TCS | | [5] |
| exon 1 | c.59delG |  | Mutation |  | TCS | | [8] |
| exon 1 | c.61G>C | p.A21P | Mutation |  | TCS | | [5] |
| exon 1 | c.77C>T | p.A26V | Mutation |  | TCS | | [9] |
| exon 1 | c.79G>A | p.A27T | Mutation |  | TCS | | [5] |
| exon 1 | c.87delA | p.V30* | Mutation | STOP | TCS | | [5] |
| exon 1 | ND | p.Q36* | Mutation | STOP | TCS | | [10] |
| intron 1 | c.108+62_68del |  | Unclassified variants |  | TCS | | [5] |
| intron 1 | c.109-1delG |  | Mutation |  | TCS | | [10] |
| intron 1 | c.109-2A>T |  | Mutation |  | TCS | | [5] |
| intron 1 | c.109-28T>C |  | Unclassified variants |  | TCS | | [5] |
| exon 2 | c.122C>T | p.A41V | Polymorphism |  | TCS | | [11] |
| exon 2 | c.149A>G | p.Y50C | Mutation |  | TCS | | [12] |
| exon 2 | c.157T>C | p.W53R | Mutation |  | TCS | | [10,13] |
| exon 2 | c.159G>A | p.W53* | Mutation | STOP | TCS | | [5] |
| exon 2 | c.163C>T | p.Q55* | Mutation | STOP | TCS | | [5,9] |
| intron 2 | 1244G>T |  | Polymorphism |  | TCS | | [4] |
| exon 3 | c.165‑1G>A |  | Mutation | STOP | TCS | | [14] |
| exon 3 | c.218insAACC | p.A73fs | Mutation |  | TCS | | [15] |
| exon 3 | c.274 delG |  | Mutation |  | TCS | | [10] |
| exon 3 | ND | p.E93* | Mutation |  | TCS | | [10] |
| exon 3 | c.303_304delCA | p.A101Afsx73 | Mutation | STOP | TCS | | [6] |
| exon 3 | c.4369_4373 | p.K1457-Efs*12 | ND | affects NLS | TCS | | [16] |
| intron 3 | c.304+5G>C | splice | Mutation |  | TCS | | [5,8,10] |
| intron 3 | c.305-1 G>A |  | Mutation |  | TCS | | [12] |
| intron 3 | c.305-52A>G |  | Polymorphism |  | TCS | | [5] |
| exon 4 | c.343delG | p.121* | Mutation | STOP | TCS | | [7] |
| exon 4 | c.357_358delAA | p.S120fs | Mutation |  | TCS | | [5] |
| exon4/intron4 | c.376delAAGGTGAGTGGGACTGCC |  | ND | STOP | TCS | | [17] |
| intron 4 | c.379-2A>G |  | Mutation |  | TCS | | [7] |
| exon 5 | c.381_382delAG | p.A127* | Mutation | STOP | TCS | | [18] |
| exon 5 | c.389insA |  | Mutation |  | TCS | | [8] |
| exon 5 | c.390delGA | p.173* | Mutation | STOP | TCS | | [7] |
| exon 5 | c.405_406delTG | p.G136fs | Mutation |  | TCS | | [5,10,19] |
| exon 5 | c.408delG | p.214* | Mutation |  | TCS | | [7] |
| exon 5 | c.422insA | p.H141Q | Mutation | STOP | TCS | | [20] |
| exon 5 | c.431delC | p.T144fs | Mutation |  | TCS | | [15] |
| exon 5 | c.475A>T | p.K159* | Mutation | STOP | TCS | | [9] |
| exon 5 | c.484_668ins |  | ND | STOP | TCS | | [21] |
| exon 5 | c.489delC | p.S164Qfs*55 | Mutation |  | TCS | | [22] |
| exon 5 | c.497delATAC | p.N166I | Mutation | STOP | TCS | | [20] |
| exon 5 | c.503C>T | p.T168M | Polymorphism |  | TCS | | [7] |
| exon 5 | c.519delT | p.T173Tfsx46 | Mutation | STOP | TCS | | [6] |
| intron 5 | c.565+1G>A |  | Mutation |  | TCS | | [5] |
| intron 5 | c.565+18G>C |  | Polymorphism |  | TCS | | [7] |
| intron 5 | c.566-10C>A |  | Mutation |  | TCS | | [5] |
| exon 6 | c.574del(16bp) | p.209* | Mutation | STOP | TCS | | [7] |
| exon 6 | c.579G>A | p.A193A | Unclassified variants |  | TCS | | [5] |
| exon 6 | c.599delG | p.S200Tfsx19 | Mutation | STOP | TCS | | [6] |
| exon 6 | c.618delC | p.S207fs | Mutation |  | TCS | | [5] |
| exon 6 | c.630 delAG |  | Mutation |  | TCS | | [10] |
| intron 6 | c.639+1G>A |  | Mutation |  | TCS | | [15] |
| intron 6 | c.639+32C>G |  | Polymorphism |  | TCS | | [6,23] |
| intron 6 | c.639+32C>T¹ |  | Polymorphism |  | TCS | | [7,23] |
| intron 6 | c.640-69T>C |  | Polymorphism |  | TCS | | [5] |
| intron 6 | c.14036G>A |  | Polymorphism |  | TCS | | [4] |
| exon 6A | c.648delC | p.S217Qfs*2 | Mutation |  | TCS | | [22] |
| exon 6A | c.726delT | p.K244fs | Mutation |  | TCS | | [13] |
| exon 6A | c.827_844del | p.G276_E281del | Mutation |  | TCS | | [5] |
| exon 7 | IVS6-710 C-T |  | Mutation |  | TCS | | [24] |
| exon 7 | c.698delC |  | Mutation |  | TCS | | [8] |
| exon 7 | c.720-727delAGCACCCC |  | Mutation |  | TCS | | [12,24] |
| exon 7 | c.724insC |  | Mutation |  | TCS | | [10] |
| exon 7 | c.728insC | p.271* | Mutation | STOP | TCS | | [7] |
| exon 7 | c.744insT |  | Mutation | STOP | TCS | | [25] |
| exon 7 | ND | p.Q252* | Mutation |  | TCS | | [10] |
| exon 7 | c.768 G>A | p.G256G | Polymorphism |  | TCS | | [7] |
| exon 7 | c.786delA |  | Mutation |  | TCS | | [12] |
| exon 7 | c.786delAG |  | Mutation | STOP | TCS | | [25] |
| exon 7 | c.790_791delAG | p.S264QfsX7 | Mutation |  | TCS | | [26] |
| exon 7 | c.797G>A | p.S266N | Mutation |  | TCS | | [7] |
| exon 7 | c.803A>G¹ | p.E268G | Polymorphism |  | TCS | | [23] |
| exon 7 | c.1016C>G | p.S339* | Mutation | STOP | TCS | | [5] |
| exon 7 | c.1028G>A | p.S343N | Unclassified variants |  | TCS | | [5] |
| exon 7 | c.1347T>C | p.P439L | Polymorphism |  | TCS | | [6,20] |
| intron 7 | c.852+39A>G |  | Polymorphism |  | TCS | | [7] |
| intron 7 | c.1083+39G>A |  | Polymorphism |  | TCS | | [5] |
| exon 8 | c.864-865delAG |  | Mutation |  | TCS | | [8] |
| exon 8 | c.911C>T | p.S304L | Mutation |  | TCS | | [27] |
| exon 8 | c.998 C>T¹ | p.S333L | Polymorphism | affects ATM phoshorylation site | TCS | | [23] |
| exon 8 | c.1015 G>T |  | Mutation |  | TCS | | [12] |
| exon 8 | c.1086G>A | p.A362A | Unclassified variants |  | TCS | | [5] |
| exon 8 | c.1095_1096delAG | p.G366fs | Mutation |  | TCS | | [8,15] |
| exon 8 | c.1096G>T | p.G366* | Mutation | STOP | TCS | | [5] |
| exon 8 | c.1242C>T | p.S414S | Unclassified variants |  | TCS | | [5] |
| intron 8 | c.1047+60 G>C |  | Polymorphism |  | TCS | | [7] |
| intron 8 | c.1278+60G>C |  | Polymorphism |  | TCS | | [5] |
| exon 9 | c.1084G>A | p.A362T | Mutation |  | TCS | | [28] |
| exon 9 | c.1098delC |  | Mutation |  | TCS | | [12] |
| exon 9 | c.1099A>T | p.K367* | Mutation | STOP | TCS | | [7] |
| exon 9 | c.1106insC |  | Mutation | affects ATM phoshorylation site | TCS | | [8] |
| exon 9 | c.1120delG |  | Mutation |  | TCS | | [10] |
| exon 9 | c.1142delC |  | Mutation |  | TCS | | [29] |
| exon 9 | c.1150G>A, 3987insG | p.A350A | Mutation |  | TCS | | [23] |
| exon 9 | c.1164insT |  | Mutation |  | TCS | | [23] |
| exon 9 | c.1215insA |  | Mutation |  | TCS | | [12] |
| exon 9 | c.1216insA |  | Mutation |  | TCS | | [10] |
| exon 9 | c.1230A>G | p.E410E | Polymorphism |  | TCS | | [7] |
| exon 9 | c.1247A >G | p.E419E | Polymorphism |  | TCS | | [28] |
| exon 9 | c.1281G>A | p.A427A | Polymorphism |  | TCS | | [5] |
| exon 9 | c.1298delC | p.A433fs | Mutation |  | TCS | | [15] |
| exon 9 | c.1303delC | p.Q435fs | Mutation |  | TCS | | [5] |
| exon 9 | c.1446dupA | p.D483fs | Mutation |  | TCS | | [5,12,19] |
| exon 9 | c.1473dupC | p.M492fs | Mutation |  | TCS | | [5] |
| exon 10 | c.1287insG |  | Mutation |  | TCS | | [23] |
| exon 10 | c.1316C>T | p.P439L | Polymorphism |  | TCS | | [10,20,23] |
| exon 10 | c.1327insA |  | Mutation |  | TCS | | [10] |
| exon 10 | c.1347T>C¹ | p.P449P | Mutation/Polymorphism |  | TCS | | [7,20,23,24,28,30] |
| exon 10 | c.1359G>A | p.G453G | Mutation |  | TCS | | [28] |
| exon 10 | c.1393 C>T | p.Q465* | Mutation | affects NLS | TCS | | [29] |
| exon 10 | c.1406-1409delAGAG |  | Mutation |  | TCS | | [23,24] |
| exon 10 | c.1408-1409delAG | p.S470Q | Mutation | STOP | TCS | | [12,20,23,24] |
| exon 10 | c.1441insGG |  | Mutation |  | TCS | | [10] |
| exon 10 | c.1557dupA | p.G520fs | Mutation |  | TCS | | [5,31] |
| exon 10 | c.1578 T > C | p.P526P | Mutation/Polymorphism | Affects PolI interaction site | TCS | | [6,13,20] |
| exon 10 | c.1581delG | p.G587Gfsx69 | Mutation | STOP/Affects PolI interaction site | TCS | | [6] |
| exon 10 | c.1609C>T | p.Q537* | Mutation | STOP/Affects PolI interaction site | TCS | | [15] |
| exon 10 | c.1639_1640delAG | p.S547fs | Mutation | Affects PolI interaction site | TCS | | [5,6,12,20,32] |
| exon 10 | c.1702C>T | p.Q568* | Mutation | STOP/Affects PolI interaction site | TCS | | [5] |
| intron 10 | c.1473+68C>T |  | Polymorphism |  | TCS | | [7] |
| exon 11 | c.1530G>T | p.G510G | Polymorphism | Affects PolI interaction site | TCS | | [7,23,28,30] |
| exon 11 | c.1552G>A | p.V518I | Polymorphism | Affects PolI interaction site | TCS | | [7,20,23] |
| exon 11 | c.[1552delG; 1565T< | p.518* | Mutation | STOP/Affects PolI interaction site | TCS | | [7] |
| exon 11 | c.1611G>A | p.S537S | Mutation/Polymorphism | Affects PolI interaction site | TCS | | [7,20,23,24,28,30] |
| exon 11 | c.1729C>T | p.Q577* | Mutation | STOP/Affects PolI interaction site | TCS | | [5] |
| exon 11 | c.1747C>T |  | Mutation | STOP/Affects PolI interaction site | TCS | | [33] |
| exon 11 | c.1761G>T | p.G587G | Polymorphism | Affects PolI interaction site | TCS | | [6,23] |
| exon 11 | c.1762C>G¹ | p.P588A | Mutation | Affects PolI interaction site | TCS | | [30] |
| exon 11 | c.1782_1788del7 | p.Val595fs | Mutation | Affects PolI interaction site | TCS | | [5] |
| exon 11 | c.1813_1814delAT | p.M605fs | Mutation | Affects PolI interaction site | TCS | | [5] |
| exon 11 | c.1837delG | p.E613fs | Mutation | Affects PolI interaction site | TCS | | [5] |
| exon 11 | c.1842A>G | p.S614S | Polymorphism | Affects PolI interaction site | TCS | | [6,20] |
| exon 11 | c.1854G>A | p.A618A | Unclassified variants | Affects PolI interaction site | TCS | | [5] |
| exon 11 | c.17681C>T | p.P526P | Polymorphism | Affects PolI interaction site | TCS | | [4] |
| exon 11 | c.17693G>A | p.G530G | Polymorphism | Affects PolI interaction site | TCS | | [4] |
| exon 12 | c.1687C>T | p.Q563* | Mutation | STOP/Affects PolI interaction site | TCS | | [7] |
| exon 12 | c.1719delG | p.N574Tfs | Mutation | Affects PolI interaction site | TCS | | [29] |
| exon 12 | c.1742insC | p.620* | Mutation | STOP/Affects PolI interaction site | TCS | | [7] |
| exon 12 | c.1762C>G | p.P588A | Polymorphism | Affects PolI interaction site | TCS | | [7,28] |
| exon 12 | c.1768insC | p.620* | Mutation | STOP/Affects PolI interaction site | TCS | | [7] |
| exon 12 | c.1837G > C | p.A588P | Polymorphism | Affects PolI interaction site | TCS | | [6] |
| exon12 | c.1863A>G, 2355-2364delCAGGGCCAGA | p.E621E | Mutation | Affects PolI interaction site | TCS | | [23] |
| exon 12 | c.1866-1873delAGATAGTG |  | Mutation | Affects PolI interaction site | TCS | | [23] |
| exon 12 | c.1867-1868delGA | p.D623X | Mutation | STOP/Affects PolI interaction site | TCS | | [9,23] |
| exon 12 | c.1868delATAG | p.632* | Mutation | STOP/Affects PolI interaction site | TCS | | [7] |
| exon 12 | c.1872-1875delTGAG |  | Mutation | Affects PolI interaction site | TCS | | [12] |
| exon 12 | c.1879 delGAGAA |  | Mutation | Affects PolI interaction site | TCS | | [10] |
| exon 12 | c.1907delT | p.L636fs | Mutation | Affects PolI interaction site | TCS | | [5] |
| exon 12 | c.1952_1956delCTGCA | p.T651fs | Mutation | Affects PolI interaction site | TCS | | [5] |
| exon 12 | c.1953C>T | p.T651T | Unclassified variants | Affects PolI interaction site | TCS | | [5] |
| exon 12 | c.1973delC | p.P658Lfsx53 | Mutation | STOP/Affects PolI interaction site | TCS | | [6] |
| exon 12 | c.1993C>G | p.A665P | Polymorphism | Affects PolI interaction site | TCS | | [6] |
| exon 12 | c.1993delG | p.A665fs | Mutation | Affects PolI interaction site | TCS | | [5] |
| exon 12 | c.1999dupC | p.R667fs | Mutation | Affects PolI interaction site | TCS | | [5,7] |
| exon 12 | c.2065_2075del11 | p.P689fs | Mutation | Affects PolI interaction site | TCS | | [5] |
| exon 12 | c.2098_2099delGA | p.D700* | Mutation | STOP/Affects PolI interaction site | TCS | | [5,23] |
| exon 12 | c.2099_2102delATAG | p.D700fs | Mutation | Affects PolI interaction site | TCS | | [5,7] |
| exon 12 | c.2103_2106delTGAG | p.S701fs | Mutation | Affects PolI interaction site | TCS | | [5,12] |
| exon 12 | c.18111A>G | p.S614S | Polymorphism | Affects PolI interaction site | TCS | | [4] |
| intron 12 | c.1911+1G>A |  | Mutation |  | TCS | | [7] |
| intron 12 | c.1911+36delC |  | Polymorphism |  | TCS | | [7] |
| intron 12 | c.2142+22C>T |  | Unclassified variants |  | TCS | | [5] |
| exon 13 | c.1611G>A |  | Mutation | Affects PolI interaction site | TCS | | [24] |
| exon 13 | c.18434G>C | p.A665P | Polymorphism | Affects PolI interaction site | TCS | | [4] |
| exon 13 | c.1915-1916delAA |  | Mutation | Affects PolI interaction site | TCS | | [8] |
| exon 13 | c.1926-1927insG |  | Mutation | Affects PolI interaction site | TCS | | [12] |
| exon 13 | c.1974G>C¹ | p.L658F | Polymorphism | Affects PolI interaction site | TCS | | [23] |
| exon 13 | c.1978delC |  | Mutation | STOP/Affects PolI interaction site | TCS | | [21] |
| exon 13 | c.2014insG |  | Mutation | Affects PolI interaction site | TCS | | [10] |
| exon 13 | c.2014C>T¹ | p.P672S | Polymorphism | Affects PolI interaction site | TCS | | [23] |
| exon 13 | c.2018-2025delCAGTCACC |  | Mutation | Affects PolI interaction site | TCS | | [23,24] |
| exon 13 | c.2019-2025delAGTCACC |  | Mutation | Affects PolI interaction site | TCS | | [8] |
| exon 13 | c.2026C>T | p.Q676* | Mutation | STOP; affects ATM phosphorylation site, Affects PolI interaction site | TCS | | [8,12] |
| exon 13 | c.2055delAG |  | Mutation | STOP/Affects PolI interaction site | TCS, AMD? | | [34] |
| exon 13 | c.2059delAG |  | Mutation | STOP; Affects PolI interaction site | TCS | | [25] |
| exon 13 | c.2082-2085delTGAG |  | Mutation | Affects PolI interaction site | TCS | | [8] |
| exon 13 | c.2157dupG | p.K720fs | Mutation | Affects PolI interaction site | TCS | | [5,12] |
| exon 13 | c.2164delC | p.L722fs | Mutation | Affects PolI interaction site | TCS | | [5] |
| exon 13 | c.2167C>T | p.Q723* | Mutation | STOP/Affects PolI interaction site | TCS | | [5] |
| exon 13 | c.2285_2286delCT | p.S762* | Mutation | STOP/Affects PolI interaction site | TCS | | [5] |
| exon 13 | c.2285_2286delCT | p.S762fs | Mutation | STOP/Affects PolI interaction site | TCS | | [6] |
| exon 13 | c.2287G>T | p.E763* | Mutation | STOP/Affects PolI interaction site | TCS | | [5] |
| intron 13 | c.2341-2A>G |  | Mutation |  | TCS | | [5] |
| exon 14 | c.2110delG |  | Mutation | Affects PolI interaction site | TCS | | [8] |
| exon 14 | c.2221C> T | p.Q741* | Mutation | STOP/Affects PolI interaction site | TCS | | [28] |
| exon 14 | ND | p.K748K | Mutation | Affects PolI interaction site | TCS | | [19,23] |
| exon 14 | ND | p.K749K | Mutation | Affects PolI interaction site | TCS | | [10] |
| exon 14 | c.2205insTT; 2206delG | p.795* | Mutation | STOP/Affects PolI interaction site | TCS | | [7] |
| exon 14 | c.2426delC | p.P809fs | Mutation | Affects PolI interaction site | TCS | | [5] |
| exon 14 | c.2473_2476delTCCA | p.S825fs | Mutation | Affects PolI interaction site | TCS | | [5] |
| exon 14 | c.2478G>A |  | Mutation | Affects PolI interaction site | TCS | | [13,15,19,23] |
| intron 14 | c.2247+27G>A |  | Polymorphism |  | TCS | | [7] |
| intron 14 | c.2248-42insG |  | Polymorphism |  | TCS | | [7] |
| intron 14 | c.2478+5G>C |  | Mutation |  | TCS | | [5] |
| intron 14 | c.2478+5G>A |  | Mutation |  | TCS | | [22] |
| exon 15 | c.2272C>T | p.Q758* | Mutation | STOP/Affects PolI interaction site | TCS | | [8] |
| exon 15 | c.2297delG |  | Mutation | Affects PolI interaction site | TCS | | [10] |
| exon 15 | c.2354-2363delCAGGGCCAGA |  | Mutation | Affects PolI interaction site | TCS | | [23] |
| exon 15 | c.2355-2356delAG |  | Mutation | Affects PolI interaction site | TCS | | [23] |
| exon 15 | c.2375-2376delGG |  | Mutation | Affects PolI interaction site | TCS | | [12] |
| exon 15 | c.238delG |  | Mutation | Affects PolI interaction site | TCS | | [10] |
| exon 15 | c.2394_2395delAG | p.D799Qfs* | Mutation | STOP | TCS | | [18] |
| exon 15 | c.2399delGTGA |  | Mutation | Affects PolI interaction site | TCS | | [10] |
| exon 15 | c.2399-2402delGTGA |  | Mutation | Affects PolI interaction site | TCS | | [8] |
| exon 15 | ND | p.E796* | Mutation | STOP/Affects PolI interaction site | TCS | | [23] |
| exon 15 | c.2626_2627delGA | p.D876Qfsx2 | Mutation | STOP/Affects PolI interaction site | TCS | | [6] |
| intron 15 | c.2428-20insCTCT |  | Polymorphism |  | TCS | | [20,23] |
| intron 15 | c.2428-20insCTCTC |  | Polymorphism |  | TCS | | [7,20] |
| intron 15 | c.2428-36A>C |  | Polymorphism |  | TCS | | [7] |
| intron 15 | c.2659-28delTCTC |  | Polymorphism |  | TCS | | [6] |
| exon 16 | c.2429C>T¹ | p.A810V | Mutation/Polymorphism | Affects PolI interaction site | TCS | | [7,10,20,23,28,30] |
| exon 16 | c.2442delG |  | Mutation | Affects PolI interaction site | TCS | | [10] |
| exon 16 | c.2452C>T | p.Q818* | Mutation | STOP/Affects PolI interaction site | TCS | | [7] |
| exon 16 | c.2490delC | p.PR830–831PG² | Mutation | STOP/Affects PolI interaction site | TCS | | [20] |
| exon 16 | c.2526insA | p.854* | Mutation | STOP/Affects PolI interaction site | TCS | | [7] |
| exon 16 | c.2526delAG | p.TG842–843TA² | Mutation | STOP/Affects PolI interaction site | TCS | | [20] |
| exon 16 | c.2527insA |  | Mutation | Affects PolI interaction site | TCS | | [23] |
| exon 16 | c.2529ins G |  | Mutation | STOP/Affects PolI interaction site | TCS | | [25] |
| exon 16 | c.2534C>T¹ | p.S845L | Polymorphism | Affects PolI interaction site | TCS | | [20,23] |
| exon 16 | c.2542C>T | p.G848* | Mutation | STOP/Affects PolI interaction site | TCS | | [7] |
| exon 16 | c.2545delG | p.872* | Mutation | STOP/Affects PolI interaction site | TCS | | [7] |
| exon 16 | c.2552delA; 2561delA | p.K851S | Mutation | STOP/Affects PolI interaction site | TCS | | [20] |
| exon 16 | c.2565delAG | p.SG855–856SE² | Mutation | STOP/Affects PolI interaction site | TCS | | [20] |
| exon 16 | c.2622insT |  | Mutation | Affects PolI interaction site | TCS | | [8] |
| exon 16 | c.2660C>T | p.A887V | Polymorphism | Affects PolI interaction site | TCS | | [6,20] |
| exon 16 | c.2683C>T | p.Q895* | Mutation | STOP/Affects PolI interaction site | TCS | | [5,7] |
| exon 16 | c.2713G>T | p.E905* | Mutation | STOP/Affects PolI interaction site | TCS | | [5] |
| exon 16 | c.2762C>T | p.P921L | Unclassified variants | Affects PolI interaction site | TCS | | [5] |
| exon 16 | c.2765C>T | p.S992L | Polymorphism | Affects PolI interaction site | TCS | | [6,20] |
| exon 16 | c.2831delA | p.E944Efsx6 | Mutation | Affects PolI interaction site | TCS | | [6] |
| intron 16 | c.2628+26A>G |  | Polymorphism |  | TCS | | [7] |
| intron 16 | c.2629-1G>A |  | Mutation |  | TCS | | [7] |
| intron 16 | c.2629-1G>C |  | Mutation |  | TCS | | [12] |
| intron 16 | c.2629-3A>G |  | Mutation |  | TCS | | [7] |
| intron 16 | c.2859+26A>G |  | Unclassified variants |  | TCS | | [5] |
| intron 16 | c.2859-30G>A |  | Polymorphism |  | TCS | | [6] |
| intron 16 | c.2860-27G>A |  | Polymorphism |  | TCS | | [5] |
| intron 16 | c.21761-21765delCTCTC |  | Polymorphism |  | TCS | | [4] |
| exon 16A | IVS16a +C/T |  | ND |  | TCS | | [30] |
| exon 16A | c.2919G>T | p.R973S | Unclassified variants |  | TCS | | [5] |
| intron 16A | exon 16A+4C>T |  | Polymorphism |  | TCS | | [7] |
| intron 16A | c.2859+3444C > T |  | Polymorphism |  | TCS | | [6] |
| exon 17 | c.2629G>A | p.V877M | Mutation |  | TCS | | [7] |
| exon 17 | c.2643C>T | p.P881P | Polymorphism |  | TCS | | [7] |
| exon 17 | c.2643C>T | p.P881P | Polymorphism |  | TCS | | [28] |
| exon 17 | c.2731C>T | p.R911* | Mutation | STOP | TCS | | [25,33] |
| exon 17 | c.2740G>T | p.S914* | Mutation | STOP | TCS | | [8] |
| exon 17 | c.2800C>T | p.Q934* | Mutation | STOP; affects ATM phosphorylation site | TCS | | [7] |
| exon 17 | c.2802A>G | p.Q934Q | Polymorphism |  | TCS | | [28] |
| exon 17 | c.2924C>T | p.P975L | Polymorphism |  | TCS | | [6] |
| exon 17 | c.2962C>T | p.R988* | Mutation | STOP | TCS | | [5] |
| exon 17 | c.2969C>A | p.S990* | Mutation | STOP | TCS | | [5] |
| exon 17 | c.21786T>C | p.V887A | Polymorphism |  | TCS | | [4] |
| exon 17 | c.21968G>T | p.A948S | Polymorphism |  | TCS | | [4] |
| intron 17 | c.2815+1delG |  | Mutation |  | TCS | | [12] |
| intron 17 | c.3046+1G>A |  | Mutation |  | TCS | | [5] |
| intron 17 | c.3047-1G>A |  | Mutation |  | TCS | | [5] |
| intron 17 | c.3047-2A>T |  | Mutation |  | TCS | | [5] |
| intron 17 | c.3047-2A>G |  | Mutation |  | TCS | | [22] |
| exon 18 | c.2822-2823delGA |  | Mutation |  | TCS | | [23] |
| exon 18 | c.2846delC |  | Mutation |  | TCS | | [12] |
| exon 18 | c.2881delG |  | Mutation |  | TCS | | [12] |
| exon 18 | c.2952G>T | p.Q984H | Mutation | affects ATM phoshorylation site | TCS | | [25] |
| exon 18 | c.3053_3054delGA | p.R1018fs | Mutation |  | TCS | | [15,23] |
| exon 18 | c.3112delG | p.A1038fs | Mutation |  | TCS | | [5,12] |
| exon 18 | c.3118_3119dupG | p.A1040Gfx47 | Mutation |  | TCS | | [6] |
| exon 18 | c.3156C>T |  | Mutation |  | TCS | | [5] |
| exon 18 | c.3163C>T | p.Q1055* | Mutation | STOP | TCS | | [5] |
| exon 18 | c.3183>4A |  | Mutation |  | TCS | | [5] |
| exon 19 | c.2998delC |  | Mutation |  | TCS | | [10] |
| exon 19 | c.3066+59C>T, 2355delCAGGGCCAGA |  | Mutation |  | TCS | | [23] |
| exon 19 | c.3156C>T | p.G1052G | ND | STOP | TCS | | [35] |
| intron 19 | c.3066+66C>T¹ |  | Polymorphism |  | TCS | | [7,23] |
| intron 19 | c.3067-72A>C |  | Polymorphism |  | TCS | | [7] |
| intron 19 | c.3197+66C>T |  | Polymorphism |  | TCS | | [6] |
| exon 20 | c.3084delA |  | Mutation |  | TCS | | [12] |
| exon 20 | c.3086-3092delCACTCCC |  | Mutation |  | TCS | | [23] |
| exon 20 | c.3100delA |  | Mutation |  | TCS | | [23] |
| exon 20 | ND | p.Q1041* | Mutation | STOP | TCS | | [10] |
| exon 20 | c.3169C>T | p.Q1057* | Mutation | STOP | TCS | | [7] |
| exon 20 | c.3279C>T¹ | p.H1093H | Polymorphism |  | TCS | | [23] |
| exon 20 | c.3314delG | p.G1105fs | Mutation |  | TCS | | [5] |
| exon 20 | c.3456_63delTTCTTCAG | p.S1152Rfsx3 | Mutation | STOP | TCS | | [6] |
| intron 20 | c.3287-34G>A |  | Polymorphism |  | TCS | | [7] |
| intron 20 | c.3517-34G>A |  | Polymorphism |  | TCS | | [6] |
| exon 21 | c.2103_2106delTGAG | p.S701fs | Mutation |  | TCS | | [12,15] |
| exon 21 | c.3276G>C | p.R1099P | Polymorphism |  | TCS | | [28] |
| exon 21 | c.3296C>G | p.P1099R | Polymorphism |  | TCS | | [7,23] |
| exon 21 | c.3311delC |  | Mutation | Affects TOPBP1 interaction site | TCS | | [23] |
| exon 21 | c.3527C>G | p.P1176R | Polymorphism |  | TCS | | [6,23] |
| exon 21 | c.3528C>T | p.P1176P | Unclassified variants |  | TCS | | [5] |
| intron 21 | IVS21-3C/T |  | ND |  | TCS | | [30] |
| intron 21 | c.3600+1delG |  | Mutation | Affects TOPBP1 interaction site | TCS | | [5] |
| intron 21 | c.3600+64dupT |  | Unclassified variants |  | TCS | | [5] |
| intron 21 | c.3370-3C>T |  | Polymorphism |  | TCS | | [6,7,12] |
| exon 22 | c.3376-3380delCTCTC |  | Mutation |  | TCS | | [8] |
| exon 22 | c.3389T>A | p.M1130K | Mutation |  | TCS | | [12] |
| exon 22 | c.3447T>C | p.D1149D | Polymorphism |  | TCS | | [28] |
| exon 22 | c.3498T>C | p.*1168Q | Polymorphism |  | TCS | | [28] |
| exon 22 | c.3611C>G | p.S1204* | Mutation | STOP | TCS | | [5] |
| exon 22 | c.3612A>C |  | Mutation | loss of exon 22 | TCS | | [11] |
| exon 22 | c.3613G>A |  | Mutation |  | TCS | | [5] |
| exon 22 | c.3700_3704delACTCT | p.T1234Gfsx5 | Mutation |  | TCS | | [6] |
| exon 22 | c.3745_3747delinsA | p.P1249fs | Mutation |  | TCS | | [5] |
| intron 22 | c.3550+1G-A |  | Mutation |  | TCS | | [10] |
| intron 22 | c.3781+2dupT |  | Mutation |  | TCS | | [5] |
| intron 22 | c.3781+8A>G |  | Unclassified variants |  | TCS | | [5] |
| exon 23 | c.3606delAG |  | Mutation |  | TCS | | [10] |
| exon 23 | c.3639delG |  | Mutation |  | TCS | | [12,24] |
| exon 23 | c.3700delG |  | Mutation |  | TCS | | [8] |
| exon 23 | c.3711-3712delAG |  | Mutation |  | TCS | | [23] |
| exon 23 | c.3779insC |  | Mutation |  | TCS | | [12] |
| exon 23 | c.3789G>A | p.K1263K | Mutation |  | TCS | | [28] |
| exon 23 | c.3802C>A¹ | p.R1268S | Polymorphism |  | TCS | | [23] |
| exon 23 | c.3819G>A¹ | p.S1273S | Polymorphism |  | TCS | | [23] |
| exon 23 | c.3823delC | p.R1275fs | Mutation |  | TCS | | [5] |
| exon 23 | c.3830delC |  | Mutation |  | TCS | | [12] |
| exon 23 | c.3853delC | p.Q1285fs | Mutation |  | TCS | | [15] |
| exon 23 | c.3853dupC | p.Q1285fs | Mutation |  | TCS | | [5,15] |
| exon 23 | c.3876 delCGGGGAAGGTGGGGAGGCCTCTGTT and insTTC |  | Mutation | STOP | TCS | | [25] |
| exon 23 | c.3899delT |  | Mutation |  | TCS | | [12] |
| exon 23 | c.3933insG |  | Mutation |  | TCS | | [12,24] |
| exon 23 | c.3938C>T¹ | p.A1313V | Mutation/Polymorphism |  | TCS | | [7,10,20,23,24] |
| exon 23 | c.3938T>C | p.V1313A | Polymorphism |  | TCS | | [28] |
| exon 23 | c.3942A>C | p.S1314S | Unclassified variants |  | TCS | | [5] |
| exon 23 | c.3975-3979delGAAAG |  | Mutation |  | TCS | | [12] |
| exon 23 | c.3983_3986delAGAA | p.K1328fs | Mutation |  | TCS | | [5] |
| exon 23 | c.3987insG | p.1352* | Mutation | STOP | TCS | | [7,12,23] |
| exon 23 | c.4019-4032delCAGAAGAGGAGCTT |  | Mutation |  | TCS | | [12] |
| exon 23 | c.4061G>C¹ | p.G1354A | Polymorphism |  | TCS | | [7,23] |
| exon 23 | c.4061delC | p.P1354fs | Mutation |  | TCS | | [12,15] |
| exon 23 | c.4108delAA |  | Mutation |  | TCS | | [10] |
| exon 23 | c.4111+5G>C |  | Mutation |  | TCS | | [29] |
| exon 23 | c.4127_4128delCT | p.S1376fs | Mutation |  | TCS | | [5] |
| exon 23 | c.4131_4132delTT | p.S1378fs | Mutation |  | TCS | | [5] |
| exon 23 | c.4177_4178delGA | p.D1393fs | Mutation | Affects NLS | TCS | | [5] |
| exon 23 | c.4218dupG | p.S1407fs | Mutation |  | TCS | | [5,7,12,13,15,23] |
| exon 23 | c.4219delT | p.S1407fs | Mutation |  | TCS | | [5] |
| exon 23 | c.4231C>T | p.Q1411* | Mutation | STOP; affects ATM phosphorylation site | TCS | | [3,5] |
| exon 23 | c.4295_4296AT>GA | p.D1432G | Unclassified variants |  | TCS | | [5] |
| exon 23 | c.4321delG | p.E1441fs | Mutation | Affects NLS | TCS | | [5] |
| exon 23 | c.4339_4340delAA | p.K1447fs | Mutation | Affects NLS | TCS | | [5,10] |
| exon 23 | c.4342A>T | p.R1448* | Mutation | STOP; affects NLS | TCS | | [5] |
| intron 23 | c.4112-17T>A |  | Polymorphism |  | TCS | | [7] |
| exon 23B | c.4169C>T | p.A1390V | Polymorphism |  | TCS | | [6,23] |
| exon 23B | c.4292G>C | p.G1431A | Polymorphism |  | TCS | | [6,23] |
| exon 23B | c.4331C>T | p.Q1411* | Mutation | STOP; affects ATM phosphorylation site | TCS | | [3,6] |
| exon 24 | IVS24+350C/G |  | Mutation |  | TCS | | [24] |
| exon 24 | c.4130delAAAAA |  | Mutation |  | TCS | | [10] |
| exon 24 | c.4130-4134delAAAAA |  | Mutation |  | TCS | | [12] |
| exon 24 | c.4131_4135delAAAAG | p.K1380Efs* | Mutation | STOP | TCS | | [18] |
| exon 24 | c.4131-4139delAAAAGAAAA | p.1378K-1380Kdel | Mutation |  | TCS | | [9] |
| exon 24 | c.4122delCA | p.1392* | Mutation | STOP; affects NLS | TCS | | [7] |
| exon 24 | c.4124-4125delAA |  | Mutation |  | TCS | | [12] |
| exon 24 | c.4134delA |  | Mutation |  | TCS | | [12] |
| exon 24 | c.4135 del GAAAA |  | Mutation | STOP | TCS | | [8,10,12,23-25] |
| exon 24 | c.4135-4136insG |  | Mutation |  | TCS | | [12] |
| exon 24 | c.4138_4142del | p.K1380 | Mutation | STOP | TCS | | [36] |
| exon 24 | c.4148insGAA | p.insK1382 | Polymorphism |  | TCS | | [7] |
| exon 24 | c.4156-4157delAA |  | Mutation |  | TCS | | [12] |
| exon 24 | c.4344dupA | p.R1448fs | Mutation | Affects NLS | TCS | | [15] |
| exon 24 | c.4355_4356ins14 | p.1456Thrfs*18 | Mutation | STOP | TCS | | [37] |
| exon 24 | c.4359_4363delAAAAA | p.E1453Efsx16 | Mutation |  | TCS | | [6,10] |
| exon 24 | c.4361_4365delAAAAA | p.K1454fs | Mutation | Affects NLS | TCS | | [10,12,15] |
| exon 24 | c.4362_4365delAAAA | p.E1456fs | Mutation | Affects NLS | TCS | | [5] |
| exon 24 | c.4363_4365delinsTAG | p.K1455* | Mutation | STOP; affects NLS | TCS | | [5] |
| exon 24 | c.4365delA | p.E1456fs | Mutation | Affects NLS | TCS | | [5,12] |
| exon 24 | c.4366_4369delGAAA | p.E1456fs | Mutation | Affects NLS | TCS | | [5] |
| exon 24 | c.4366_4370delGAAAA | p.K1457fs | Mutation | Affects NLS | TCS | | [6,10,12,13,23] |
| exon 24 | c.4369_4373delAAGAA | p.K1457fs | Mutation | Affects NLS | TCS | | [5,8,10,12,23,25,37] |
| exon 24 | c.4375_4377delAAG | p.K1459del | Mutation | Affects NLS | TCS | | [15] |
| exon 24 | c.4377_4379delGAA | p.K1460del | Unclassified variants | Affects NLS | TCS | | [5] |
| exon 24 | c.38922C>T | p.A1390V | Polymorphism |  | TCS | | [4] |
| intron 24 | IVS24+350C>G |  | Mutation |  | TCS | | [24] |
| intron 24 | IVS24+439C>A |  | Mutation |  | TCS | | [24] |
| intron 24 | c.4209+42C>A |  | Polymorphism |  | TCS | | [7] |
| intron 24 | c.4440+106G>T |  | Unclassified variants |  | TCS | | [5] |
| intron 24 | c.4440+108C>A |  | Polymorphism |  | TCS | | [5] |
| exon 25 | c.4224 G>A, 2822-2823delGA | p.E1508E | Mutation |  | TCS | | [23] |
| ND | c.2757_2758delAG | p.G920fs | Mutation |  | TCS | | [13,20] |

1. Larsen, D.H.; Hari, F.; Clapperton, J.A.; Gwerder, M.; Gutsche, K.; Altmeyer, M.; Jungmichel, S.; Toledo, L.I.; Fink, D.; Rask, M.B.; et al. The NBS1-Treacle complex controls ribosomal RNA transcription in response to DNA damage. *Nat Cell Biol* **2014**, *16*, 792-803, doi:10.1038/ncb3007.

2. Marsh, K.L.; Dixon, J.; Dixon, M.J. Mutations in the Treacher Collins syndrome gene lead to mislocalization of the nucleolar protein treacle. *Hum Mol Genet* **1998**, *7*, 1795-1800, doi:10.1093/hmg/7.11.1795.

3. Korsholm, L.M.; Gál, Z.; Lin, L.; Quevedo, O.; Ahmad, D.A.; Dulina, E.; Luo, Y.; Bartek, J.; Larsen, D.H. Double-strand breaks in ribosomal RNA genes activate a distinct signaling and chromatin response to facilitate nucleolar restructuring and repair. *Nucleic Acids Res* **2019**, *47*, 8019-8035, doi:10.1093/nar/gkz518.

4. Hao, S.; Jin, L.; Wang, H.; Li, C.; Zheng, F.; Ma, D.; Zhang, T. Mutational Analysis of TCOF1, GSC, and HOXA2 in Patients With Treacher Collins Syndrome. *J Craniofac Surg* **2016**, *27*, e583-586, doi:10.1097/SCS.0000000000002934.

5. Bowman, M.; Oldridge, M.; Archer, C.; O'Rourke, A.; McParland, J.; Brekelmans, R.; Seller, A.; Lester, T. Gross deletions in TCOF1 are a cause of Treacher-Collins-Franceschetti syndrome. *Eur J Hum Genet* **2012**, *20*, 769-777, doi:10.1038/ejhg.2012.2.

6. Conte, C.; D'Apice, M.R.; Rinaldi, F.; Gambardella, S.; Sangiuolo, F.; Novelli, G. Novel mutations of TCOF1 gene in European patients with Treacher Collins syndrome. *BMC Med Genet* **2011**, *12*, 125, doi:10.1186/1471-2350-12-125.

7. Teber, O.A.; Gillessen-Kaesbach, G.; Fischer, S.; Böhringer, S.; Albrecht, B.; Albert, A.; Arslan-Kirchner, M.; Haan, E.; Hagedorn-Greiwe, M.; Hammans, C.; et al. Genotyping in 46 patients with tentative diagnosis of Treacher Collins syndrome revealed unexpected phenotypic variation. *Eur J Hum Genet* **2004**, *12*, 879-890, doi:10.1038/sj.ejhg.5201260.

8. Dixon, J.; Ellis, I.; Bottani, A.; Temple, K.; Dixon, M.J. Identification of mutations in TCOF1: Use of molecular analysis in the pre- and postnatal diagnosis of Treacher Collins syndrome. *American Journal of Medical Genetics Part A* **2004**, *127A*, 244-248, doi:10.1002/ajmg.a.30010.

9. Fujioka, H.; Ariga, T.; Horiuchi, K.; Ishikiriyama, S.; Oyama, K.; Otsu, M.; Kawashima, K.; Yamamoto, Y.; Sugihara, T.; Sakiyama, Y. Detection of a novel silent deletion, a missense mutation and a nonsense mutation in TCOF1. *Pediatr Int* **2008**, *50*, 806-809, doi:10.1111/j.1442-200X.2008.02650.x.

10. Edwards, S.J.; Gladwin, A.J.; Dixon, M.J. The mutational spectrum in Treacher Collins syndrome reveals a predominance of mutations that create a premature-termination codon. *Am J Hum Genet* **1997**, *60*, 515-524.

11. Macaya, D.; Katsanis, S.H.; Hefferon, T.W.; Audlin, S.; Mendelsohn, N.J.; Roggenbuck, J.; Cutting, G.R. A synonymous mutation in TCOF1 causes Treacher Collins syndrome due to mis-splicing of a constitutive exon. *Am J Med Genet A* **2009**, *149A*, 1624-1627, doi:10.1002/ajmg.a.32834.

12. Splendore, A.; Jabs, E.W.; Passos-Bueno, M.R. Screening of TCOF1 in patients from different populations: confirmation of mutational hot spots and identification of a novel missense mutation that suggests an important functional domain in the protein treacle. *J Med Genet* **2002**, *39*, 493-495, doi:10.1136/jmg.39.7.493.

13. Splendore, A.; Fanganiello, R.D.; Masotti, C.; Morganti, L.S.; Passos-Bueno, M.R. TCOF1 mutation database: novel mutation in the alternatively spliced exon 6A and update in mutation nomenclature. *Hum Mutat* **2005**, *25*, 429-434, doi:10.1002/humu.20159.

14. Yan, Z.; Lu, Y.; Wang, Y.; Zhang, X.; Duan, H.; Cheng, J.; Yuan, H.; Han, D. Identification of a novel TCOF1 mutation in a Chinese family with Treacher Collins syndrome. *Exp Ther Med* **2018**, *16*, 2645-2650, doi:10.3892/etm.2018.6446.

15. Masotti, C.; Ornelas, C.C.; Splendore-Gordonos, A.; Moura, R.; Félix, T.M.; Alonso, N.; Camargo, A.A.; Passos-Bueno, M.R. Reduced transcription of TCOF1 in adult cells of Treacher Collins syndrome patients. *BMC Med Genet* **2009**, *10*, 136, doi:10.1186/1471-2350-10-136.

16. Giabicani, E.; Lemale, J.; Dainese, L.; Boudjemaa, S.; Coulomb, A.; Tounian, P.; Dubern, B. Chronic intestinal pseudo-obstruction in a child with Treacher Collins syndrome. *Arch Pediatr* **2017**, *24*, 1000-1004, doi:10.1016/j.arcped.2017.07.004.

17. Marszalek, B.; Wisniewski, S.A.; Wojcicki, P.; Kobus, K.; Trzeciak, W.H. Novel mutation in the 5' splice site of exon 4 of the TCOF1 gene in the patient with Treacher Collins syndrome. *Am J Med Genet A* **2003**, *123A*, 169-171, doi:10.1002/ajmg.a.20312.

18. Li, X.; Su, Y.; Huang, S.; Gao, B.; Zhang, D.; Wang, X.; Gao, Q.; Pang, H.; Zhao, Y.; Yuan, Y.; et al. Genotype-phenotype variability in Chinese cases of Treacher Collins syndrome. *Acta Oto-Laryngologica* **2019**, *139*, 567-575, doi:10.1080/00016489.2019.1612530.

19. Gladwin, A.J.; Dixon, J.; Loftus, S.K.; Edwards, S.; Wasmuth, J.J.; Hennekam, R.C.; Dixon, M.J. Treacher Collins syndrome may result from insertions, deletions or splicing mutations, which introduce a termination codon into the gene. *Hum Mol Genet* **1996**, *5*, 1533-1538, doi:10.1093/hmg/5.10.1533.

20. Wise, C.A.; Chiang, L.C.; Paznekas, W.A.; Sharma, M.; Musy, M.M.; Ashley, J.A.; Lovett, M.; Jabs, E.W. TCOF1 gene encodes a putative nucleolar phosphoprotein that exhibits mutations in Treacher Collins Syndrome throughout its coding region. *Proc Natl Acad Sci U S A* **1997**, *94*, 3110-3115, doi:10.1073/pnas.94.7.3110.

21. Marszałek-Kruk, B.A.; Wójcicki, P.; Smigiel, R.; Trzeciak, W.H. Novel insertion in exon 5 of the TCOF1 gene in twin sisters with Treacher Collins syndrome. *J Appl Genet* **2012**, *53*, 279-282, doi:10.1007/s13353-012-0091-3.

22. Fan, X.; Wang, Y.; Fan, Y.; Du, H.; Luo, N.; Zhang, S.; Chen, X. TCOF1 pathogenic variants identified by Whole-exome sequencing in Chinese Treacher Collins syndrome families and hearing rehabilitation effect. *Orphanet J Rare Dis* **2019**, *14*, 178, doi:10.1186/s13023-019-1136-z.

23. Splendore, A.; Silva, E.O.; Alonso, L.G.; Richieri-Costa, A.; Alonso, N.; Rosa, A.; Carakushanky, G.; Cavalcanti, D.P.; Brunoni, D.; Passos-Bueno, M.R. High mutation detection rate in TCOF1 among Treacher Collins syndrome patients reveals clustering of mutations and 16 novel pathogenic changes. *Hum Mutat* **2000**, *16*, 315-322, doi:10.1002/1098-1004(200010)16:4<315::AID-HUMU4>3.0.CO;2-H.

24. Splendore, A.; Jabs, E.W.; Félix, T.M.; Passos-Bueno, M.R. Parental origin of mutations in sporadic cases of Treacher Collins syndrome. *Eur J Hum Genet* **2003**, *11*, 718-722, doi:10.1038/sj.ejhg.5201029.

25. Horiuchi, K.; Ariga, T.; Fujioka, H.; Kawashima, K.; Yamamoto, Y.; Igawa, H.; Sugihara, T.; Sakiyama, Y. Mutational analysis of the TCOF1 gene in 11 Japanese patients with Treacher Collins Syndrome and mechanism of mutagenesis. *Am J Med Genet A* **2005**, *134*, 363-367, doi:10.1002/ajmg.a.30357.

26. Schlump, J.U.; Stein, A.; Hehr, U.; Karen, T.; Möller-Hartmann, C.; Elcioglu, N.H.; Bogdanova, N.; Woike, H.F.; Lohmann, D.R.; Felderhoff-Mueser, U.; et al. Treacher Collins syndrome: clinical implications for the paediatrician--a new mutation in a severely affected newborn and comparison with three further patients with the same mutation, and review of the literature. *Eur J Pediatr* **2012**, *171*, 1611-1618, doi:10.1007/s00431-012-1776-7.

27. Papageorgiou, E.; Papoulidis, I.; Zavlanos, A.; Papanikolaou, E.; Manolakos, E.; Fidani, S. A novel familial mutation associated with Treacher Collins syndrome: A case report. *Biomed Rep* **2020**, *12*, 285-289, doi:10.3892/br.2020.1284.

28. Su, P.H.; Yu, J.S.; Chen, J.Y.; Chen, S.J.; Li, S.Y.; Chen, H.N. Mutations and new polymorphic changes in the TCOF1 gene of patients with oculo-auriculo-vertebral spectrum and Treacher-Collins syndrome. *Clin Dysmorphol* **2007**, *16*, 261-267, doi:10.1097/MCD.0b013e3281c108d2.

29. Zhang, C.; An, L.; Xue, H.; Hao, S.; Yan, Y.; Zhang, Q.; Jin, X.; Li, Q.; Zhou, B.; Feng, X.; et al. Mutation analysis of TCOF1 gene in Chinese Treacher Collins syndrome patients. *J Clin Lab Anal* **2020**, e23567, doi:10.1002/jcla.23567.

30. Thiel, C.T.; Rosanowski, F.; Kohlhase, J.; Reis, A.; Rauch, A. Exclusion of TCOF1 mutations in a case of bilateral Goldenhar syndrome and one familial case of microtia with meatal atresia. *Clin Dysmorphol* **2005**, *14*, 67-71, doi:10.1097/00019605-200504000-00003.

31. The Treacher Collins Syndrome Collaborative, G.; Dixon, J.; Edwards, S.J.; Gladwin, A.J.; Dixon, M.J.; Loftus, S.K.; Bonner, C.A.; Koprivnikar, K.; Wasmuth, J.J. Positional cloning of a gene involved in the pathogenesis of Treacher Collins syndrome. *Nature Genetics* **1996**, *12*, 130-136, doi:10.1038/ng0296-130.

32. Shoo, B.A.; McPherson, E.; Jabs, E.W. Mosaicism of a TCOF1 mutation in an individual clinically unaffected with Treacher Collins syndrome. *Am J Med Genet A* **2004**, *126A*, 84-88, doi:10.1002/ajmg.a.20488.

33. Horiuchi, K.; Ariga, T.; Fujioka, H.; Kawashima, K.; Yamamoto, Y.; Igawa, H.; Sakiyama, Y.; Sugihara, T. Treacher Collins syndrome with craniosynostosis, choanal atresia, and esophageal regurgitation caused by a novel nonsense mutation in TCOF1. *Am J Med Genet A* **2004**, *128A*, 173-175, doi:10.1002/ajmg.a.30038.

34. Goverdhan, S.V.; Temple, I.K.; Self, J.; Lotery, A.J.; Dixon, M.J.; Evans, A.R. Macular degeneration associated with a novel Treacher Collins tcof1 mutation and evaluation of this mutation in age related macular degeneration. *Br J Ophthalmol* **2005**, *89*, 1063-1064, doi:10.1136/bjo.2004.064139.

35. Wilbe, M.; Gudmundsson, S.; Johansson, J.; Ameur, A.; Stattin, E.L.; Annerén, G.; Malmgren, H.; Frykholm, C.; Bondeson, M.L. A novel approach using long-read sequencing and ddPCR to investigate gonadal mosaicism and estimate recurrence risk in two families with developmental disorders. *Prenat Diagn* **2017**, *37*, 1146-1154, doi:10.1002/pd.5156.

36. Kantaputra, P.N.; Tripuwabhrut, K.; Intachai, W.; Carlson, B.M.; Quarto, N.; Ngamphiw, C.; Tongsima, S.; Sonsuwan, N. Treacher Collins syndrome: A novel TCOF1 mutation and monopodial stapes. *Clinical Otolaryngology* **2020**, *45*, 695-702, doi:10.1111/coa.13560.

37. Bauer, M.; Saldarriaga, W.; Wolfe, S.A.; Beckwith, J.B.; Frias, J.L.; Cohen, M.M. Two extraordinarily severe cases of Treacher Collins syndrome. *Am J Med Genet A* **2013**, *161A*, 445-452, doi:10.1002/ajmg.a.35397.
