# Supplementary material for: The Role of TCOF1 Gene in Health and Disease: Beyond Treacher Collins Syndrome
Source: Int J Mol Sci. 2021 Mar 1;22(5):2482. doi: 10.3390/ijms22052482 (PMC7957619; doi:10.3390/ijms22052482)
Supplement: Supplementary file 1 [file ijms-22-02482-s001.zip › Sup/Figure S1, S2.doc]

**Figure S1.** The figure shows the transcripts encoding the TCOF1 protein. The boxes represent the exons coding for the N-terminal region (green boxes on the left), the central domain (red boxes and an additional blue one that indicates exon 6A) and the N-terminal domains of treacle (green boxes on the right). The predicted treacle protein variants can range in size from 188aa to 1489aa. Ensembl ([https://www.ensembl.org](https://www.ensembl.org/)) transcripts’ IDs are given on the left of each transcript.

k

**Figure S2.** The role of treacle in response of cancer cells to DNA damaging agents. (**A)** Following irradiation-induced DNA damage treacle is phosphorylated by DDR kinases ATM/ATR, as well as CK2 [5, 34], resulting in phosphobinding motifs which enable interaction with NBS1. This in turn results in treacle-mediated nucleolar localization of NBS1 which forms MRN complex at the sites of DNA breaks. In addition, treacle phosphorylation enables binding and recruitment of TOPBP1 to rDNA breaks [5]. Moreover, treacle and TOPBP1 mediate nucleolar accumulation of ATR, the central kinase required for repair of rDNA breaks [5]. ATR activity leads to repression of Pol I transcription and the broken rDNA is transported out of the nucleoli to the nucleolar caps enabling accession of DSB repair proteins. Cells treated with ATM/ATR/CK2 inhibitors **(B)** or devoid of treacle (e.g. due to siRNA-mediated treacle depletion) **(C)** are unable to recruit key factors required for DDR, which leads to accumulation of DNA breaks and induction of apoptosis.
